# Implications of Breeding for Growth on Drought Tolerance in Scots Pine ( *Pinus sylvestris* L.)—Insights From Metabolomics and High‐Throughput Plant Architecture Analysis

**DOI:** 10.1111/eva.70122

**Published:** 2025-06-23

**Authors:** Francisco Gil‐Muñoz, Sonali Sachin Ranade, Haleh Hayatgheibi, Juha Niemi, Lars Östlund, María Rosario García‐Gil

**Affiliations:** ^1^ Umeå Plant Science Centre (UPSC), Department of Forest Genetics and Plant Physiology Swedish University of Agricultural Sciences Umeå Sweden; ^2^ Faculty of Forest Sciences, Department of Forest Ecology and Management Swedish University of Agricultural Sciences Umeå Sweden

**Keywords:** abscisic acid, drought, metabolomics, root architecture, scots pine

## Abstract

Drought has been identified as one of the important environmental factors in the context of climate change due to its interaction with other biotic and abiotic stresses. However, only a few studies have reported the effect of breeding on forest adaptability to climate change. Using a common garden experiment with seedlings from families of Scots pine ( *Pinus sylvestris* L.) from northern Sweden, we have found differences in drought tolerance between seedlings from breeding stands and those from natural forests. We performed a genetic analysis including high‐throughput image‐based phenotyping of seedling canopy and root traits and conducted metabolomic and hormone analyses with the aerial parts of the seedlings. Our results indicate that root architecture traits associated with drought tolerance exhibit moderate to high heritability. Analyses of seedling architecture reveal that families from breeding stands have higher drought resistance but lower genetic variation than the ones from natural forests, especially in the case of canopy traits. Metabolomic and hormone analyses of the aerial parts of the seedlings also support that the breeding stands may have a higher capacity to withstand or deal with drought conditions as compared to the natural forests. For example, increase in abscisic acid along with increase in tryptophan and auxin conjugates in the breeding stands compared to the natural forests under drought conditions may contribute to alleviation of drought response in the breeding stands. The methodology employed to evaluate drought tolerance and plant architecture in this study might be useful for future research and forest management focused on climate change adaptability.

## 1 Introduction

Droughts, windstorms, and floods are becoming more frequent and severe as a result of climate change worldwide (Spinoni et al. 2018) including northern Europe (Venäläinen et al. 2020). Drought increases the susceptibility of forests to storm and wind damage (Csilléry et al. 2017) and is becoming one of the main causes for forestry loss in Europe (Senf et al. 2020). Therefore, with the increase in the frequency of drought events (Spinoni et al. 2018), integrating stress tolerance into forest management and breeding is becoming gradually more important in order to maintain forests (Dale et al. 2001).

In the last decade, research on morphological drought response in plants has grown at an exponential rate. Studies conducted to investigate drought tolerance in various plant species have revealed that root characteristics such as main root diameter (Richards et al. 2001), percentage of fine roots (Fitter 2002) and root density/surface (Turner et al. 2001) are associated with improved performance during water stress (Wasaya et al. 2018), particularly in drought‐prone and low‐productivity areas (Kuijken et al. 2015). For example, plants with deep and fine roots exhibit better drought tolerance, as deeper roots are able to reach deeper water reserves (Pirtel et al. 2021) and fine roots are less prone to cavitation (Phillips et al. 2016). In conifers, needle morphology, and root and branching architecture have been linked with drought tolerance (Baldi and La Porta 2022; Gebauer et al. 2015, 2019; Moran et al. 2017). Other studies have even suggested needle lifespan during drought stress as a key factor for drought tolerance (Song et al. 2022).

Root shape adaptation has received a lot of attention since the origin of plant science (Cannon 1911; Hales 1727; Wasaya et al. 2018). However, due to the complexity of the root traits and technical challenges, investigating root characteristics remains difficult (Nielsen et al. 1997; Sharma and Carena 2016). According to Lynch (1995), root system architecture is divided into four different aspects: morphology, topology, distribution, and architecture. Morphology refers to the surface features of a single root axis, topology refers to how roots are connected from a branching perspective, distribution refers to the presence of root positional gradients, and architecture refers to the spatial configuration of the root system or the explicit deployment of root axes.

Although attempts have been made to incorporate root characteristics as a selection criterion in tree improvement programs (Baldi and La Porta 2022), the difficulties in assessing root properties have hampered them. In conifers, breeding for drought tolerance has primarily focused on individual traits such as root depth (Cregg and Zhang 2001; Kolb et al. 2016; Matías et al. 2014) or general plant growth (de la Mata et al. 2014; Kerr et al. 2015), whereas efforts to improve drought tolerance based on root and/or canopy architecture are yet to be made. Similarly, the fact that root‐related traits are affected by high genotype by environment interaction (G × E) (Orman‐Ligeza et al. 2014) due to factors such as temperature (Nagel et al. 2009), water availability (Bengough et al. 2011), and nutrients (Paterson et al. 2006; Pearse et al. 2006), poses an additional challenge (Kuijken et al. 2015). However, the use of phenotyping platforms and experimental designs to account for environmental effects has been proposed to improve heritability estimates by decreasing the sampling error (Kuijken et al. 2015).

Scots pine ( *Pinus sylvestris* L.) is one of the most economically important forest tree species in Fennoscandia. The tree improvement program for Scots pine in Sweden was initiated in the early 1950s by selecting around 1300 plus‐trees from natural stands based on their superior phenotypes. In the early 1980s, an additional 4700 plus‐trees were selected, forming the base material that was used to establish the founder populations in the long‐term Scots pine breeding program in Sweden for the selection of superior trees which are exploited as seed donors and grafts in seed orchards (Andersson et al. 2003). A large proportion of commercial stands are regenerated with seeds collected from superior trees that have been optimised for growth. Previously, an unfavourable correlation between growth and wood density in Scots pine (Hong et al. 2014) and other conifer species (e.g., Chen et al. 2016) has been documented. A decrease in wood density has been linked to higher vulnerability to cavitation and thus increased susceptibility to drought in forest tree species (Hentschel et al. 2014; Rosner 2017). Larger trees have also been described as having greater water demand, which may result in structural overshoot, exacerbating forests' susceptibility to drought (Liang et al. 2021). Given these considerations, it is reasonable to question whether breeding for enhanced growth has impacted these forests' tolerance to drought and capacity to adapt to future increases in drought intensity. Finding early selection traits regarding root or canopy related to improved drought tolerance will be of great interest for achieving optimal yield under adverse conditions, which will benefit the forest industry. In our study, we performed a greenhouse common garden experiment with Scots pine seedlings from two different sources, natural unmanaged forests and breeding stands. The seedlings were treated with controlled and drought conditions as a proxy to investigate the effect of breeding on tolerance and resistance to drought. High‐throughput analysis, including image‐based phenotyping, was carried out on canopy and root traits. In addition, metabolomic and hormone analyses with the aerial parts of the seedlings were conducted to detect the metabolites involved in drought tolerant or drought sensitive individuals.

Metabolomic profiling of drought resistance and tolerance has been investigated extensively in flowering plants, especially in model species like *Arabidopsis thaliana* and crops (Fabregas and Fernie 2019; Zhang et al. 2024). Drought resistance in trees denotes keeping its maximum economic yield under limited water conditions, meanwhile tolerance implies survival with low tissue water content (Panda et al. 2021). Metabolites including amino acids like proline and gamma‐aminobutyric acid (GABA), and hormones such as abscisic acid (ABA) were discussed as classical examples of drought response as their accumulation was correlated with drought tolerance in these reports. In conifers, the regulation of metabolites linked to drought tolerance has been investigated in several *Pinus* species, including Scots pine, *Pinus taeda* , *Pinus pinaster* , *Pinus halepensis* and *Pinus massoniana* (Lauder et al. 2019). Particularly in Scots pine, alterations in the secondary metabolites in response to drought have been reported in the needles and roots (Hunziker et al. 2024; Sancho‐Knapik et al. 2017). However, these cited research investigations have studied natural populations, and the impact of breeding for better yield on drought tolerance is not known, despite having very important implications. Previous studies have reported that hormonal responses to short‐term and long‐term water deficit in Scots pine and Norway spruce trees from natural forests result in increased ABA under long‐term water deficit in both species (Pashkovskiy et al. 2022). However, it has been shown that neither ABA nor cytokinins regulate stomatal conductance in Scots Pine during post‐drought recovery (Zlobin et al. 2023). In the current work, we are interested in investigating the role of metabolic profiles to determine the differences in drought tolerance between breeding stands and natural forests of Scots pine. We performed gas chromatography–mass spectrometry (GC–MS), which allows identification and quantification of the metabolites involved in the primary metabolic pathways such as sugars, sugar alcohols, amino acids, organic acids and polyamines (Schauer and Fernie 2006). In addition, hormone analysis was carried out to elucidate the differential alteration of hormones in response to drought, as hormones form essential components of plant growth and development (Yoshida and Fernie 2024).

## 2 Material and Methods

### 2.1 Plant Material

Cones were collected from a total of 60 Scots pine trees, aged between 30 to 250 years, from three sites (20 trees per site) along the natural unmanaged continuous Scots pine forest in Sweden, located in western Sweden between latitudes 63°–66° N and longitudes 16°–18° E (see Figure S1 for sampling sites: Jokkmokk (Karatj‐Råvvåive 66°41′14.2″ N 18°56′37.4″ E), Arjeplog (66°18′15.8″ N 18°21′6.5″ E), and Jämtland (Källberget‐Storberget, 63°23′52.1″ N 15°28′0.6″ E) and Table S1 for trees sampled per site). Similarly, cones were collected from 60 Scots pine trees from three breeding stands (20 trees per stand) situated near the three natural sampling sites (Figure S1). The trees in the breeding stands are the result of one breeding cycle for the selection of superior trees for volume. The base breeding stand used in this breeding cycle consisted of trees visually selected from the respective natural forest sampled for this study. The seeds collected from both natural forests and breeding stands represented open‐pollinated families. Considering that the three pairs of natural forests and breeding stands share location (site), we can assume that the sampled progenies (cones) were the result of pollination by the same pollen cloud. In addition, it is worth remarking that despite the 3° difference in latitude between the two northernmost sites and the southernmost site sampled in this study, a significant signal of latitudinal adaptation to drought is not expected, yet site was considered in the statistical models. This is based on the following main arguments: first, Scots pine populations are not genetically differentiated with a global Fst of 0.04 across its whole species distribution (Bruxaux et al. 2024), and second, the three sampled sites have a similar drought index (drought index is explained in the later section under *Statistical analysis*). The sampling design was chosen to meet the main objective: to select sites where natural forests and breeding stands were located close to each other for comparative purposes, and where the natural forests were accessible and sampling permission had been granted.

### 2.2 Greenhouse Conditions and Drought Stress

Seeds extracted from cones of the 120 sampled trees were sown using a five‐block completely randomised design, with a progeny size of 12 for each treatment (drought and control), resulting in 24 seeds per sampled tree. Each block consisted of two trays per treatment, with 144 seeds per tray (1440 seeds per treatment in total). Due to the low germinability of some of the stands, the number of families and individuals per family was uneven. This is a common problem in forestry; however, this work included a sufficient number of individuals to conduct the statistical analyses with confidence. Only families with at least three individuals in both the control and drought treatments were retained for further analyses. This resulted in 11 and 24 families from natural forests and breeding stands, respectively, that were included in the subsequent analyses. The average number of individuals per family for natural forests was 10 individuals, while it was 15 individuals for breeding stands (see Table S1 for the details).

Seeds were sown in a mixture of 2:1 vol:vol of garden soil (Planteringsjord, Plantagen, Sweden) and sand (Sandlådesand, Boke, Sweden). Seeds were germinated in AirBlock 121 (BCC AB, Sweden) trays of 54 cc volume per cell. Trays were placed above a water flow restriction matrix of floral foam blocks (Oasis, USA) to simulate the water table as described in Marchin et al. (2020). A total height of 3 and 21 cm between the bottom of the tray and the water level was used as control and drought conditions, respectively. One cm of sand was placed between the foam and the trays to ensure contact between the trays and the matrix. Seeds were sown on day 1 under greenhouse conditions, where the temperature was maintained between 25°C and 35°C and a 16:8 h light: darkness photoperiod with Fiona Lightning (FL300) light from Senmatic in the sunlight mode. The seeds germinated between 12 and 23 days after sowing. The outermost cells of the trays were filled with soil, but no plants were grown in these cells to avoid the border effect. Before the drought experiment started, all the seedlings were fertilised once a week by top watering for 35 days (HORTO LIQUID Rika S 7‐1‐5 at 10 mL/L concentration). The drought and control treatments were initiated 55 days after sowing. The drought and control treatments lasted 45 days, during which the plants were not irrigated from above nor fertilised. Water availability was measured during the entire period using a tensiometer (Figure S2).

### 2.3 Image Acquisition

At the end of the experiment, plants were collected carefully, removing the soil, and placed in a custom‐built acrylic cuvette with 1 cm of water to ensure full root expansion (i.e., roots showed their natural shape) without tension, as described by York (2023). The cuvette was placed over a scanner (Epson Perfection V550), and each plant included in the experiment was scanned at 600 dpi. A blue background was applied to facilitate image segmentation. Images were cropped during preprocessing, and each scanned image was separated into root and canopy. For image segmentation, a machine‐learning approach using Ilastik software (Berg et al. 2019) was used to assess canopy and root. Probability maps generated in Ilastik were used as input for the root analysis software, which assessed both root and canopy traits. The software used for root analysis was Rhizovision Explorer (Seethepalli et al. 2021). Settings for analysis were: Whole Root, Image Thresholding Level 120, Keep Largest Component, Edge Smoothing Threshold 2, and Root Pruning Threshold 1.

### 2.4 Root and Canopy Parameters

After image acquisition, root and canopy parameters were scored for further analysis. Root parameters were scored as described by Seethepalli et al. (2021). The following traits were recorded: Median number of roots, number of root tips, total root length, root depth, root width to depth ratio, root network area, root convex area, root solidity, lower root area, root average diameter, root perimeter, root volume, root surface area, no. of root holes, average root hole size, average root orientation, root shallow angle frequency, root medium angle frequency, and root steep angle frequency as defined by Seethepalli et al. (2021) (https://www.rhizovision.com/manual).

Finally, the proportion of root surface comprised by roots with diameter < 0.25 mm, 0.25–0.5 mm, 0.5–1 mm and > 1 mm was recorded (traits Prop Surface Area Diameter Range 1, 2, 3, 4, respectively). Canopy parameters were calculated with a similar pipeline as the root parameters, and the following traits were assessed: Canopy height, maximum canopy width, canopy width to depth ratio, canopy convex area, canopy solidity, needle average diameter, needle median diameter, canopy perimeter, canopy volume, average needle orientation and canopy surface area. Total volume was the addition of root and canopy volumes calculated by the Rhizovision Explorer software. Ratios between canopy and root volume, and canopy and root surface were also calculated.

### 2.5 Statistical Analysis

Statistical analysis was performed using RStudio (R version 4.2.1, R Development Core Team 2022; RStudio version 1.4.1743‐4, RStudio Team 2021) with the following packages: dplyr (Wickham et al. 2023), ggplot2 (Wickham 2016), tidyverse (Wickham et al. 2019), corrplot (Wei and Simko 2024), stringr (Wickham 2023), factoextra (Kassambara and Mundt 2020), RcolorBrewer (Neuwirth 2014), emmeans (Lenth 2024) and car (Fox and Weisberg 2019). For the exploratory analysis, a principal component analysis (PCA) was done for root traits at the individual level of each seedling.

To calculate the drought tolerance index, a linear regression was used for the total seedling volume in drought and control conditions for each family. The index is calculated based on the studentised residuals of the single family from a mathematical regression relationship between the trait under control and drought conditions (Bidinger et al. 1982):

$$V_{d}={\mathrm{\alpha V}}_{c}+e,$$

where *V*_*d*_ is the family average of the total volume of the seedling in drought conditions, *V*_*c*_ is the family average of total volume in control conditions, *α* is the proportional reduction in volume due to drought, and *e* is the random residual effect. The deviation of each family from the model was used as a tolerance index (underperformance and overperformance in drought conditions). The effect of the source (natural or breeding) of the seeds was calculated by adding the source effect to the linear equation with interaction.

For testing the effect of family, source, and location (site of sampling), analysis was done using general linear models (GLM). Gaussian, Poisson or gamma distributions were used according to the distribution of the measured traits. To compensate for the disbalanced design, differences between effects were tested using Type III sum of squares analysis of deviance with Bonferroni corrections. We fitted the data to the following statistical models:

Family model (ANOVA F‐test)

Regarding family effect, replicates consist of a single data point per seedling in the following model:

$$Y_{\text{ijklm}}=\mu +\alpha \times P_{i}+\beta \times P_{j}+\gamma \times P_{k}+\delta \times {\mathrm{Pl}}_{l}+\varepsilon \times P_{i}\times P_{j}+e_{\text{ijkl}},$$

where $Y_{\text{ijklm}}$ is the trait phenotype on the mth seedling, μ is the overall mean of the response, $P_{i}$ is the fixed effect of theith family, $P_{j}$ is the fixed effect of the *j*th treatment: Drought ($P_{j}$ = 1) or Control ($P_{j}$ = 0), $P_{k}$ is the fixed effect of the *k*th block, ${\mathrm{Pl}}_{l}$ is the random effect of the age of the plant after germination (Days After Germination), $P_{i}$ × $P_{j}$ is the interaction effect between the *i*th family and the *j*th treatment and $e_{\text{ijkl}}$is the random residual effect. The terms *α*, *β*, *γ*, *δ* and 𝜀 correspond to the coefficients of the corresponding effects. Each trait was adjusted to its distribution type.

Source model (GLM)

Regarding source effect, replicates consist of a single data point per seedling, the model is

$$Y_{\text{ijkl}}=\mu +\alpha \times P_{i}+\beta \times P_{j}+\gamma \times P_{k}+\delta \times {\mathrm{Pl}}_{l}+\varepsilon \times P_{i}\times P_{j}+e_{\text{ijkl}},$$

where $Y_{\text{ijkl}}$ is the trait phenotype on the nth seedling, μ is the overall mean of the response, $P_{i}$ is the fixed effect of theith source: Breeding ($P_{i}$ = 1) or Natural ($P_{i}$ = 0), $P_{j}$ is the fixed effect of the *j*th treatment: Drought ($P_{j}$ = 1) or Control ($P_{j}$ = 0), $P_{k}$ is the fixed effect of the *k*th block, ${\mathrm{Pl}}_{l}$ is the random effect of the age of the plant after germination (Days After Germination), $P_{i}$ × $P_{j}$ is the interaction effect between the *i*th source and the *j*th treatment and $e_{\text{ijkl}}$is the random residual effect. The terms *α*, *β*, *γ*, *δ* and 𝜀 correspond to the coefficients of the corresponding effects. Each trait was adjusted to its distribution type.

After the full analysis, we presented in the paper only canopy and root traits that showed at least a moderate association with drought tolerance (*r* > 0.4) and low correlation between traits (*r* < 0.2) (r refers to Pearson's correlation coefficient, significance calculated based on Fischer's *Z* transformation with 95% confidence level).

### 2.6 Quantitative Genetic Parameter Estimates

Variance components were estimated for six groups of progenies categorised by their source (natural or breeding) and growing conditions (control or drought): (1) Progenies from natural forests, (2) progenies from breeding stands, (3) progenies from natural forests grown under control conditions, (4) progenies from natural forests grown under drought conditions, (5) progenies from breeding stands grown under control conditions, and (6) progenies from breeding stands grown under drought conditions.

A mixed‐linear model approach, implemented in the ASReml4 statistical software package (Gilmour et al. 2015) was used following the model below:

Y=Xβ+Zu+e,

where Y is the vector of observations; *β* is the vector of fixed effects (i.e., overall mean, days after germination, treatment, and population); u is the vector of the random additive genetic effect of individual trees, random effect of block and the interaction of additive genetic effect and the treatment; and *e* is the vector of random residual effect. X and Z are the incidence matrices relating the observations in Y to β and u, respectively. All random effects were assumed to be independently and normally distributed with the expected mean of zero where $\mathrm{var}\;\left(u\right)=A\sigma^{2}$ and $\mathrm{var}\;\left(e\right)=e^{2}$ and A is the pedigree‐based numerator relationship matrix.

Individual‐tree narrow‐sense heritability estimates ($h^{2}$) were calculated as follows:

$$h^{2}=\frac{\sigma_{A}^{2}}{\sigma_{A}^{2}+\sigma_{B}^{2}+\sigma_{\mathrm{AT}}^{2}{+\sigma }_{e}^{2}},$$

where $\sigma_{A}^{2}$, $\sigma_{B}^{2}$, $\sigma_{\mathrm{AT}}^{2}$, and $\sigma_{e}^{2}$ are the additive genetic, block, interaction between additive genetic and treatment, and error variance components, respectively. The standard errors for variance components and genetic parameters were estimated by using Taylor series approximation (Gilmour et al. 2009).

The coefficient of additive genetic variance (CVA%) was calculated as

$${\hat{\mathrm{CV}}}_{A}=\frac{\hat{\sigma_{A}}}{\bar{X}}\times 100,$$

where $\sigma_{A}$ is the standard deviation of additive genetic variance and $\bar{X}$ is the phenotypic mean of the trait.

### 2.7 Gas Chromatography–Mass Spectrometry (GC–MS) and Hormone Analysis

GC–MS allows identification and quantification of the metabolites involved in the primary metabolic pathways such as sugars, sugar alcohols, amino acids, organic acids and polyamines (Schauer and Fernie 2006). GC–MS and hormone analyses were performed with the aerial parts of seedlings, at the end of the experiment, after 45 days of drought and control treatments. The same seedlings used for root and canopy trait variation analyses were used for this purpose. GC–MS and hormone analyses were carried out including three families from the natural forests and nine families from the breeding stands. For GC–MS with natural forests, nine seedlings were treated with control conditions and eight seedlings (one sample was an outlier which was excluded from analysis) were treated with drought conditions. For GC–MS with breeding stands, 27 seedlings were treated with control conditions and 26 (one sample was an outlier which was excluded from analysis) were treated with drought conditions. For hormone analysis with natural forests, three seedlings were treated with control conditions and three seedlings (one sample was an outlier which was excluded from analysis) were treated with drought conditions. For hormone analysis with breeding stands, nine seedlings were treated with control conditions and nine seedlings (one sample was an outlier, which was excluded from analysis) were treated with drought conditions. An untargeted metabolomic approach was followed and the identification of the compounds was carried out by referring to the SMC library of authentic standards (https://www.swedishmetabolomicscentre.se/). The aerial parts of the seedlings were ground into fine powder in frozen conditions using liquid nitrogen and stored at −80 until further processing for metabolite extraction. Detailed information regarding sample preparation, mass spectrometry and data processing is included in supplementary data (Supporting Information GC–MS and Hormonomics). Statistical analyses for the detected metabolites were carried out using Soft Independent Modelling by Class Analogy (SIMCA) and t‐test.

## 3 Results

### 3.1 Effect of Drought Stress on Root and Canopy

**FIGURE 1:**
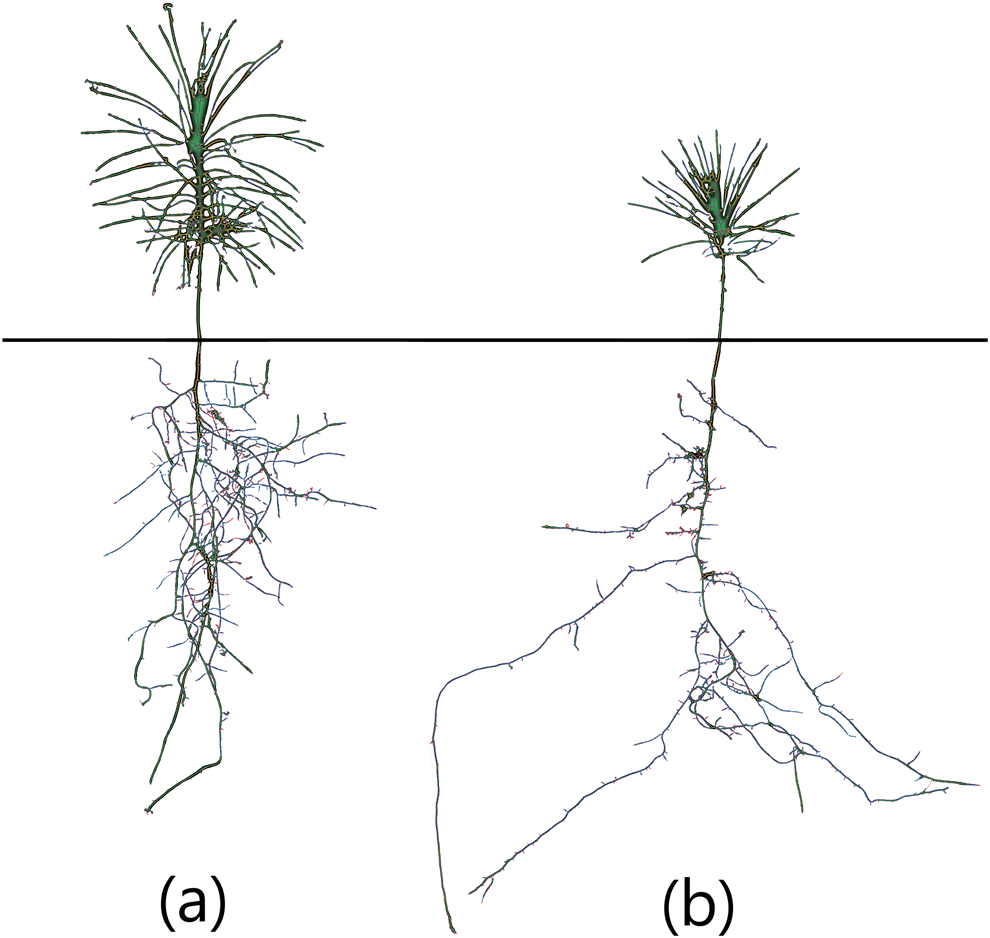
Example phenotype of plants of the same family in control (a) and drought (b) conditions. Images are segmentations of both canopies and roots of seedlings.

All the measured parameters were affected by drought except canopy solidity, needle average diameter, root convex area and root medium angle, according to the linear model (Table S2). Canopy‐related traits, such as width‐to‐depth ratio and needle angle, increased in response to drought treatment. Meanwhile, the other canopy traits showed lower values under drought. The root system tends to be smaller in both surface and volume, and less compact, but deeper in response to drought. The root system had fewer tips, and the roots were more horizontally orientated under drought compared to the control. Drought also resulted in a higher proportion of roots with smaller sections compared to the control (Table S2). The canopy‐to‐root surface and canopy‐to‐root volume decreased under drought conditions, due to a larger reduction in canopy surface and volume compared to the reduction in root surface and volume. An average response of the canopy and root system is illustrated in Figure 1.

**FIGURE 2:**
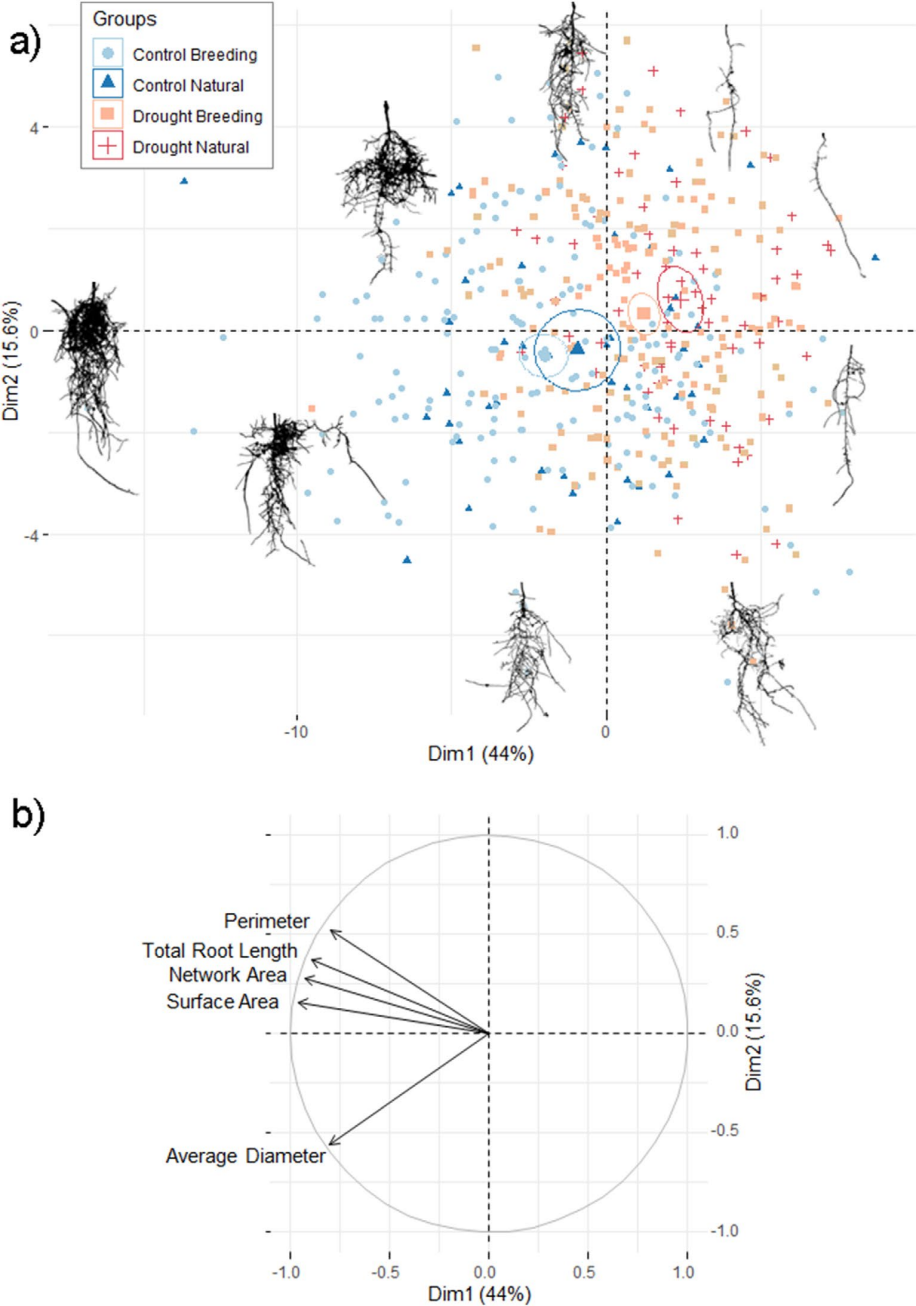
(a) Principal component analysis of root parameters in control (blue) and drought (red) conditions for each seedling. Root scans included as reference in the image correspond to the PCA coordinates for each image. Averages of natural forests and breeding stands are included with 95% confidence ellipse. (b) Vectors of the top five most contributing traits to the PCA, ordered by quality of representation (cos2).

To further investigate the effect of drought on the root system, we represented all the root‐related traits in a PCA projection for each seedling (Figure 2) under both control and drought conditions. The PC1 axis primarily reflected a gradient in root system density, ranging from denser (negative values) to less dense (positive values). Meanwhile, the PC2 axis indicated a shift in the root system structure, from an inverse pyramid‐like distribution (negative values) to a pyramid‐like structure (positive values). According to the projection, the drought response affects root shape primarily by reducing root density and altering the spatial distribution of the upper sections. In other words, the root system under control conditions appeared to have a denser arrangement of superficial roots.

### 3.2 Drought Tolerance Index

**FIGURE 3:**
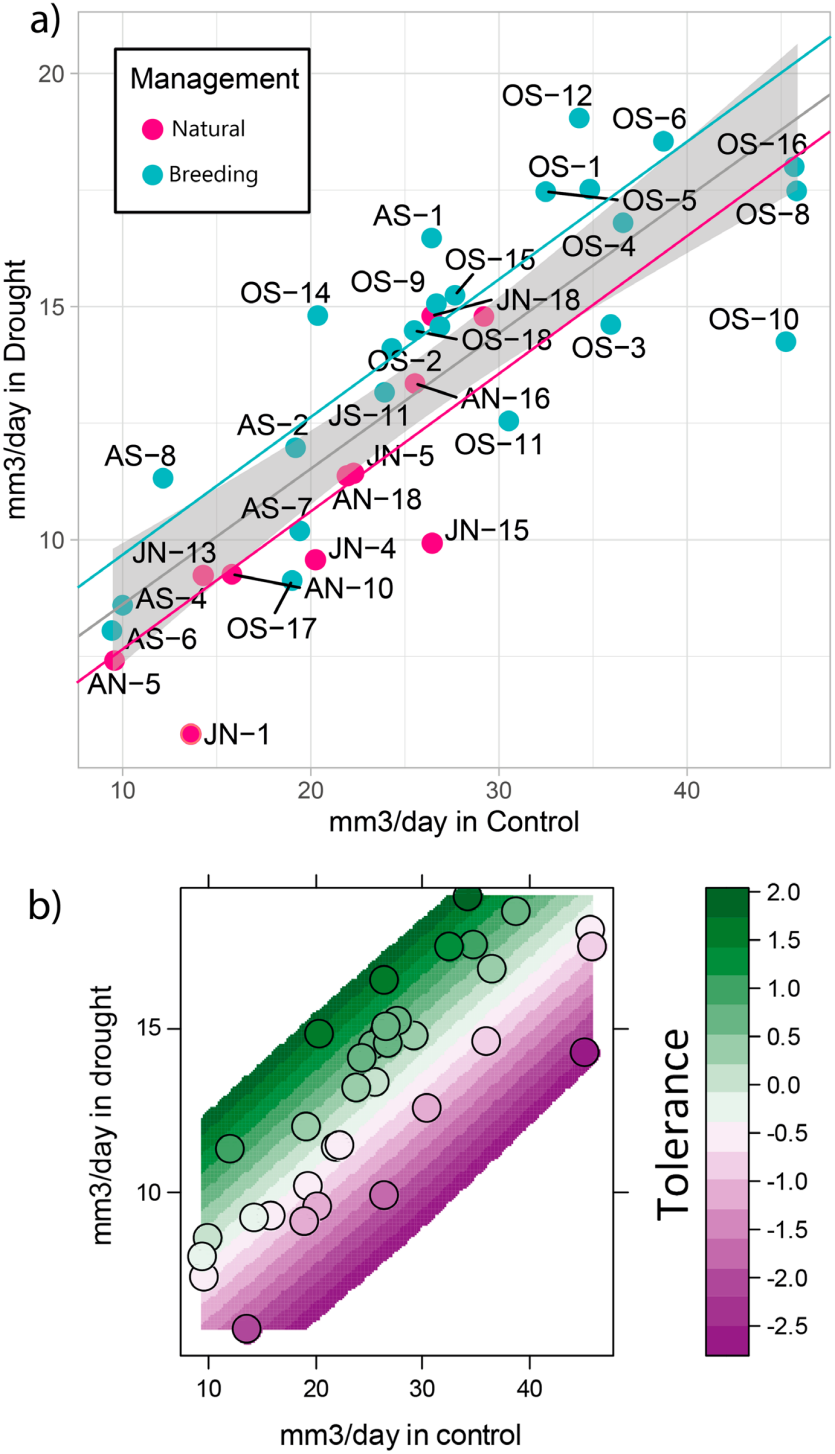
(a) Relationship between total volume (mm^3^/day, canopy and root) in control and drought conditions by family. Linear model included with 95% confidence for general average. Breeding stands model (blue) and natural forests model (magenta) are included. (b) Relationship between the total seedling volume (mm^3^/day) in control and drought conditions in two‐month‐old seedlings of different families of Scots pine. Tolerance index is calculated as deviation from the linear model. Each dot is the average measurement of a family in each condition (between 3 to 12 replicates per condition). AN, Arjeplog Natural; AS, Arjeplog Breeding; JN, Jokkmokk Natural; JS, Jokkmokk Breeding; OS, Jämtland Breeding.

To define the drought tolerance index, we performed a regression analysis between control and drought conditions using the total seedling volume (i.e., canopy and roots) obtained by scanning the plants. The analysis compared the family average daily total biomass volume between drought and control conditions. The general model for the entire population indicated that, under drought conditions, an average Scots pine seedling grows at a rate of 0.28 × Growth in control +3.6 mm^3^ per day (Figure 3). Family deviations from the linear model were used to calculate the drought tolerance index. In other words, the families that grew more than expected were considered tolerant, and those that grew less were considered susceptible as detailed in the Materials and Methods Section.

We produced correlations between family‐based tolerance index and traits measured under control conditions (Figure S3). Canopy shape, canopy width‐to‐depth ratio, and needle median diameter under control conditions were moderately correlated with the tolerance parameter (*r* < 0.4). Under drought conditions, most of the parameters showed a significant positive correlation with the tolerance index (Figure S4). Among the parameters negatively correlated under drought, the strongest correlation was found for root hole size (meaning the average area not filled with roots inside the root system hull) and root orientation angle. This indicates that families with higher tolerance indexes tend to have denser roots with smaller angles (pointing downward), while families with a high proportion of roots with diameters between 0.25 and 1 mm (Range between 2 and 3) showed a smaller drought tolerance.

### 3.3 Effect of Breeding on Tree Resilience to Drought

**FIGURE 4:**
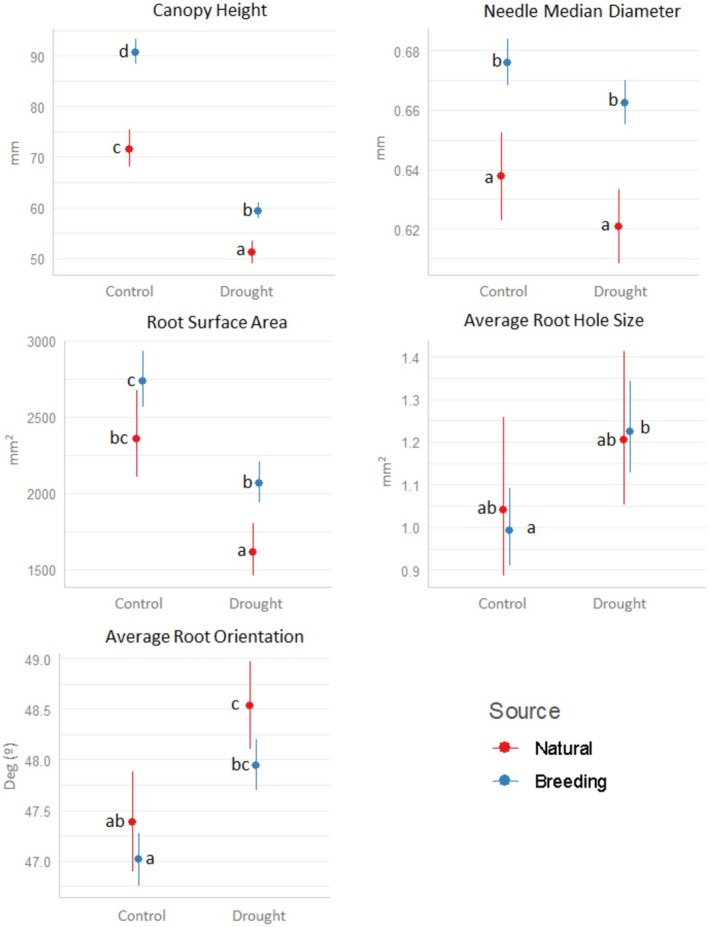
Simulated effects mean calculated from the GLM model of canopy and root related trait parameters. The parameters shown are moderately correlated (*r* > 0.4) with drought tolerance either in control or drought conditions and not highly intercorrelated (*r* < 0.2). Statistical differences between families from natural forests or breeding stands are shown both in control and drought conditions (Type III analysis of deviance with Bonferroni correction).

For the traits correlated with the drought tolerance index and without high intercorrelation, we observed that seedlings from natural forests had lower Canopy Height and Needle Median Diameter under both conditions. Seedlings from natural forests exhibited lower Root Surface Area compared to those from breeding stands, but only under drought conditions (Figure 4, Table S2). The traits, for example, average root hole size and average root orientation, did not differ between natural forests and breeding stands (Figure 4). Other canopy and root traits with at least a moderately significant correlation with drought tolerance but not presented in the main text due to trait intercorrelation also showed a statistical difference between natural forests and breeding stands (Table S2). Additionally, a higher number of traits showed significant differences between natural forests and breeding stands in canopy‐related traits compared to root traits, with a higher level of significance observed in canopy traits (Table S2).

A linear regression model approach was used to further study the effect of selection on the response of forest trees to drought (Figure 3). Populations from natural forests showed an average growth reduction of 24.5% (1.7 mm^3^/day less from 7 mm^3^/day baseline) under drought conditions compared to breeding stands, indicating a significant effect of breeding (*p* < 0.05). No interaction effect (i.e., different slope) was observed, meaning the proportional reduction of growth in drought versus control conditions was consistent across all populations. Natural forests exhibited a lower average drought tolerance index than the breeding stands.

### 3.4 Quantitative Genetic Parameter Estimates

Narrow‐sense heritability estimates ($h^{2}$) and the coefficient of additive genetic variance (CVA%), which measure genetic variation, were obtained across the six categories described in material and methods section. For simplicity, the $h^{2}$ and CVA% values for all properties are provided in Table S3 of supplementary materials and Figure S5, while the CVA% values for five traits discussed in the main text are presented in Table 1. In general, the CVA% was significantly lower for seedlings from breeding stands than for those from natural forests. For example, CVA% decreased by approximately 39%, 63%, and 6% for canopy height (mm), root surface area (mm^2^) and needle median diameter (mm), respectively, in the seedlings from breeding stands relative to natural forests. Conversely, CVA% was slightly higher for average root hole size (mm^2^) in the seedlings from breeding stands compared to natural forests (Table 1).

**TABLE 1:** Coefficient of additive genetic variance (CVA%) estimated for different properties.

Trait	Unit	Coefficient of additive genetic variance (CVA%)	
		Group	
		Breeding	Natural
Canopy height	mm	4.4	7.2
Needle median diameter	mm	4.9	5.2
root surface area	mm^2^	10.6	29.0
average root‐hole size	mm^2^	18.9	15.0
Average root orientation	Degree (Deg)	0	2.6

### 3.5 Gas Chromatography–Mass Spectrometry (GC–MS) and Hormone Analysis

The GC–MS analysis detected and identified 73 metabolites (putatively annotated) in each of the control versus drought comparisons, for example, within the seedlings from natural forests and within the seedlings from breeding stands. Multivariate analysis performed using SIMCA showed a clear separation between the control and drought samples; however, no separation was observed between the natural forests and the breeding stands with reference to both metabolite and hormone analysis (Figure S6). A comparative analysis between control and drought samples from natural forests and the breeding stands was performed separately for individual metabolites using a *t*‐test. Only the metabolites showing a significant difference (*p*‐value < 0.05) between control and drought conditions were included in the analysis, resulting in a total of 60 metabolites. Those metabolites significantly associated with drought and with an absolute fold change difference ≥ 0.5 (log2) between natural forests and breeding stands were considered the key metabolites that showed differential regulation/synthesis under drought conditions between natural forests and breeding stands (Table 2). A fold change cut‐off of ≥ 0.5 (log2) was used as this is the standard cut‐off value followed by several studies related to gene expression (Euring et al. 2021; Marzotto et al. 2014; Ranade et al. 2019; Stearns et al. 2012) and metabolomics (De Smet et al. 2016; Yang et al. 2019), allowing investigation of genes/metabolites with relatively small but biologically meaningful changes in their expression/synthesis. There were 21 metabolites that showed the absolute fold change difference ≥ 0.5 (log2) (Table 2). All these metabolites, except one (3‐oxoglutaric acid), are mainly involved in plant growth, defence and stress tolerance as reviewed and reported by earlier investigations in different plant species including conifers (Table 2). Metabolites with a fold change difference of < 0.5 (log2) between the breeding stands and natural forests were considered metabolites detected with a similar fold change in the two populations (39 metabolites, Table S4). Hormone analysis detected a total of 22 metabolites in each of the populations, out of which 10 metabolites showed an absolute fold change difference ≥ 0.5 (log2) between the control versus drought comparisons within the breeding stands and within the natural forests (Table 3). Only three metabolites related to hormones were detected with the absolute fold change difference < 0.5 (log2) between the breeding stands and natural forests (Table S5). Metabolites with non‐significant *p*‐values in both comparisons (i.e., control versus drought samples within natural forests and control versus drought samples within breeding stands) were excluded from the current analysis.

**TABLE 2:** List of metabolites detected with absolute fold change difference of 0.5 (log2) or more between the comparisons—control versus drought natural forests and control versus drought breeding stands.

Metabolite	Control versus drought breeding stands		Control versus drought natural forests		Absolute value of fold change difference between natural forests & breeding stands (log2)	Metabolite class	Function	References
	Fold change under drought (log2)	*p*‐value	Fold change under drought (log2)	*p*‐value				
Allothreonine	−1.93	0.00	−2.41	0.00	0.5	Amino acids and peptides	Stress response	Charlton et al. (2008)
Threonine	−1.84	0.00	−2.34	0.00	0.5	Amino acids and peptides	Stress response	Charlton et al. (2008)
Alpha‐tocopherol	2.57	0.00	2.08	0.00	0.5	Quinones and hydroquinones	Stress tolerance	Munné‐Bosch (2005)
Beta‐cyanoalanine	−0.89	0.03	−2.36	0.01	1.5	Amino acids and peptides	Stress/drought tolerance	Machingura et al. (2013)
Taxifolin	3.72	0.00	2.46	0.01	1.3	Flavonoids	Stress/drought tolerance	Witzell and Martín (2008)
Alanine	−1.81	0.00	−2.29	0.00	0.5	Amino acids and peptides	Defence, stress‐protective role	Parthasarathy et al. (2019)
Gamma‐aminobutyric acid (GABA)	−1.97	0.00	−2.64	0.00	0.7	Fatty acids	Defence	Guo et al. (2023)
Isoleucine	−0.49	0.01	—	Not significant	0.5	Amino acids and peptides	Defence—disease resistance	Li et al. (2021)
Isopimaric acid	1.15	0.00	2.17	0.03	1.0	Isoprenoids	Defence—negatively affects herbivory performance, increases plant resistance	Kopper et al. (2005); López‐Goldar et al. (2020)
Abietic acid	—	Not significant	1.36	0.00	1.4	Isoprenoids	Defence against fungi	Trapp and Croteau (2001)
Glutamine	−4.43	0.00	−5.46	0.00	1.0	Amino acids and peptides	Defence and metabolic fuel	Zhang et al. (2023)
Phenylalanine	−1.05	0.00	—	Not significant	1.1	Amino acids and peptides	Defence, lignin synthesis	Yadav and Chattopadhyay (2023)
Dehydroabietate	0.47	0.01	1.07	0.00	0.6	Isoprenoids	Defence, prevents insect herbivores	Hamberger et al. (2011)
3‐oxoglutaric acid	−2.69	0.00	—	Not significant	2.7	Alkaloids	Function not explored in plants	
Arabinose	1.50	0.00	2.05	0.00	0.6	Carbohydrates Monosaccharides	Growth, stress tolerance, cell wall biosynthesis	Mathisson et al. (2021)
Raffinose	2.48	0.00	—	Not significant	2.5	Carbohydrates Oligosaccharides	Growth, stress‐tolerance, maintains osmotic balance under drought stress, energy and carbon source	Hunziker et al. (2024); Wu et al. (2023)
Raffinose 2	1.49	0.00	—	Not significant	1.5	Carbohydrates Oligosaccharides	Growth, stress‐tolerance, maintains osmotic balance under drought stress, energy and carbon source	Hunziker et al. (2024); Wu et al. (2023)
Erythritol	0.60	0.02	—	Not significant	0.6	Carbohydrates Monosaccharides	Growth	Lewis and Smith (1967)
Glutamic acid	−2.56	0.00	−3.37	0.00	0.8	Amino acids and peptides	Growth and development	Liao et al. (2022)
Oxoglutaric acid	−0.79	0.00	−1.39	0.01	0.6	TCA acids	TCA—energy‐yielding metabolism	Zhang and Fernie (2018)
Succinic acid	−0.50	0.00	—	Not significant	0.5	TCA acids	TCA—energy‐yielding metabolism	Kiliç (2023)

**TABLE 3:** List of metabolites from hormone analysis detected with fold change difference of 0.5 (log2) or more between the comparisons—control versus drought natural forests and control versus drought breeding stands.

Metabolite type	Metabolite	Control versus drought breeding stands		Control versus drought natural forests		Absolute value of fold change difference between natural forests & breeding stands (log2)	Function	References
		Fold change under drought (log2)	*p*‐value	Fold change under drought (log2)	*p*‐value			
Abscisic acid	Abscisic acid	0.58	0.01	—	Not significant	0.6	Stress response	Chen et al. (2020); Takahashi et al. (2020)
Salicylic acid	Salicylic acid	−0.72	0.02	—	Not significant	0.7	Defence, stress tolerance	Khan et al. (2015)
Jasmonates	cis‐12‐oxo‐phytodienoic acid	−0.55	0.02	—	Not significant	0.6	Defence	Liu and Park (2021)
Auxin	IAA‐aspartate	—	Not significant	−1.10	0.01	1.1	Growth and development	Ludwig‐Müller (2011)
Auxin	IAA‐glucose	0.96	0.00	—	Not significant	1.0	Growth and development	Ludwig‐Müller (2011)
Auxin	IAA‐glutamate	−2.33	0.02	−3.39	0.02	1.1	Growth and development	Ludwig‐Müller (2011)
Auxin	2‐oxindole‐3‐acetic acid	1.65	0.01	—	Not significant	1.7	Growth and development	Ludwig‐Müller (2011)
Auxin	Tryptophan	3.48	0.00	—	Not significant	3.5	Auxin biosynthesis, growth and development	Mano and Nemoto (2012)
Cytokinin	Dihydrozeatin	1.10	0.00	—	Not significant	1.1	Positive/negative effects on drought stress	Cortleven et al. (2019); Tran et al. (2007)
Cytokinin	Isopentenyladenine	0.70	0.00	—	Not significant	0.7	Positive/negative effects on drought stress	Cortleven et al. (2019); Tran et al. (2007)

## 4 Discussion

Drought in Northern Europe is projected to increase as a result of climate change, particularly during the summer (Spinoni et al. 2018). However, the numerous experimental approaches to assess drought, coupled with the lack of consensus, underscore the complexity of this research topic (Munns et al. 2010). Additionally, the diversity in experimental designs may have introduced various biases, particularly in cases where the drought treatment was applied inappropriately (Marchin et al. 2020). In our study, we implemented a water restriction matrix as proposed by Marchin et al. (2020) simulating drought‐restricted water availability in Scots pine by lowering the water table, which is the closest method to mimic the water dynamics under drought in nature.

### 4.1 Effects of Drought on Seedling Traits: Beyond Linear Relationships

Our study shows that the reduction in biomass production rate is linear between drought and control conditions. Thus, the deviation from this expected linear relationship can serve as a useful indicator of drought tolerance in a controlled conditions test, as previously published (Bidinger et al. 1982). For example, a linear relationship between average needle diameter and drought tolerance under control and drought conditions could be implemented as an early selection method for drought tolerance. Although this approach could serve as an easy‐to‐apply early selection method to predict drought tolerance even under control conditions, further studies are needed to confirm its reliability across a larger number of families and populations. Previously, other studies have investigated the relationship between needle phenotype and drought tolerance. For example, needle lifespan in the tree has been previously identified as a driving factor of drought tolerance in gymnosperms (Song et al. 2022) and changes in its morphology due to drought stress have been described and linked to hydraulic properties (Gebauer et al. 2015; Grill et al. 2004). For some traits, non‐linear relationships (optimum) can obscure linear‐based models. A clear example of this is the relationship between transpiration and assimilation, where there is an optimum of transpiration at the maximum assimilation rate (Brendel 2021). According to earlier research, non‐linear models can be the best for better prediction of some traits of plants under drought stress (van der Tol, Dolman, et al. 2008; van der Tol, Meesters, et al. 2008).

### 4.2 Breeding for Increased Volume Has Enhanced Drought Tolerance

One of the key objectives in tree improvement programs is to increase biomass production per breeding cycle. Our study corroborates the positive effect of breeding on canopy and root system biomass production already at the seedling stage. Interestingly, such an effect on biomass seems to have resulted in an increase in the level of drought tolerance in breeding stands. Differences in drought tolerance between breeding generations have been previously reported. A first study in *Pinus radiata* found a provenance‐dependent relationship between breeding generation and drought tolerance, which varied as either positive or negative (Espinoza et al. 2016; Nuhu 2022). A second study reported that natural and second‐generation breeding families of coastal Douglas‐fir exhibited higher drought tolerance compared to third‐generation breeding families (Nuhu 2022). Furthermore, the same study found that plants showing robust growth under control conditions tend to have a greater ability to withstand drought, in agreement with our findings. Both this and our findings contrast with observations in other conifers, where slow growth does not necessarily correlate with drought tolerance (Csilléry et al. 2020). In coastal Douglas‐fir, for instance, no significant relationship between growth and drought tolerance was identified (Anekonda et al. 2002). These contrasting results suggest that the impact of breeding on drought tolerance may vary depending on species, population and the selection and management methods used. As a result, this relationship should be investigated on a case‐by‐case basis.

### 4.3 Enhanced Drought Tolerance in Breeding Stands Comes at the Cost of Genetic Erosion

In several species, forest management through breeding has been studied for its potential source of genetic erosion (Cortés et al. 2020; Olsson et al. 2023; Rungis et al. 2019), which largely depends on the type of management, population size and mating system (Ratnam et al. 2014). In our study, we observed a decrease in genetic diversity in the natural forests for several canopy and some root traits. This consequence of intensive selection was already observed in previous studies, which highlighted the risks of pursuing short‐term goals in species with long breeding cycles (Namkoong 1984). Our findings support that natural forests may serve as reservoirs of genetic variation. The loss of genetic diversity could be even more pronounced in advanced generation breeding programs, where growth is the primary selection criterion. This effect was previously observed in coastal Douglas‐fir, where drought tolerance was lower in the third breeding generation compared to the second (Nuhu 2022). It has already been noted that the increasing frequency of extreme climatic events may exceed the genetic adaptive capacity of forests, where the forests may struggle to keep pace with climate change (Aitken and Bemmels 2016), which is likely to be amplified in forests affected by genetic erosion.

### 4.4 Breeding for Drought Tolerance

An adaptive climate‐smart forest management can have a central role in sustaining forests in the future (Yousefpour et al. 2017). Here, we present evidence about the possibility of breeding different traits that can lead to better adaptation of Scots pine to environmental stresses and also regarding how the forest management can affect breeding potential and adaptation to present and future climatic events.

Previous studies on Scots pine features associated with needles have found heritability estimates between 0.30 to 0.88 (Donnelly et al. 2016). Additional gymnosperm species were shown to have low heritability for root‐related features such as root length (depth) and root:shoot ratio (Galeano and Thomas 2023). In our study, we found canopy traits to have high heritability estimates, while heritability values for root traits were moderate. Furthermore, some of the root multi‐trait PCA components showed moderate heritability. Similar evidence has been presented for the genetic control of a root partitioning coefficient in *Pinus pinaster* (Wu and Yeh 1997). Our study's findings on the moderate to high heritability of traits related to drought tolerance point to the potential for early selection of drought‐tolerant genotypes. This strategy is supported by the observation that conifer species' ability to withstand drought appears to depend more heavily on young plants than on mature ones (Andivia et al. 2020). Further research should be done to confirm that early‐selection traits keep tolerance in adult plants, but nevertheless, as seedling mortality can be the main limiting factor in natural populations (Castro et al. 2004), evaluating these traits can help in assessing the potential vulnerability of natural populations to drought episodes.

### 4.5 Metabolites Facilitating Drought Tolerance

There were 21 metabolites that showed differential regulation/synthesis under drought conditions between the natural forests and breeding stands (Table 2). From these 21 metabolites, 3‐oxoglutaric acid is excluded from the analysis. 3‐oxoglutaric acid is an alkaloid detected in mosses (Wang et al. 2020) and in members from the plant families Erythroxylaceae and Solanaceae (Huang et al. 2019), but its function in plants has not been explored, particularly with reference to drought. Based on the regulation of 13 out of the 20 metabolites in the breeding stands (Table 2), we propose that these stands may have a higher capacity to withstand or cope with drought conditions compared to the natural forests. All the 13 metabolites, including allothreonine, threonine, alpha‐tocopherol, beta‐cyanoalanine, taxifolin, alanine, GABA, glutamine, raffinose, raffinose 2, erythritol, glutamic acid, and oxoglutaric acid, are known to enhance drought and stress tolerance in plants. For example, although the levels of amino acids such as threonine, allothreonine, alanine, isoleucine, and glutamine are lower under drought conditions in both populations, the levels are relatively lower in the natural forests compared to the breeding stands (Table 2). These amino acids may help the breeding stands withstand the drought stress better than the natural forests, as suggested by earlier investigations. For instance, the accumulation of valine/isoleucine under drought stress is known to aid drought tolerance in angiosperms (Bowne et al. 2012; Joshi et al. 2010); alanine, the precursor to β‐alanine, is recognised for its stress‐protective role in plants (Parthasarathy et al. 2019) and threonine/glutamine play roles in plant defence (Charlton et al. 2008; Zhang et al. 2023). There are a few more metabolites that may similarly enhance the breeding stands' drought tolerance as their levels are relatively lower in the natural forests compared to the breeding stands: glutamic acid, an amino acid involved in plant growth (Liao et al. 2022); beta‐cyanoalanine, which is involved in the response to and tolerance of water deficit (Machingura et al. 2013); and GABA, which plays a role in plant responses to biotic and abiotic stresses (Guo et al. 2023). Alpha‐tocopherol, taxifolin, and isopimaric acid were found to be upregulated in natural forests and breeding stands under drought, although levels of alpha‐tocopherol and taxifolin, both involved in stress tolerance (Munné‐Bosch 2005; Witzell and Martín 2008), were relatively higher in the breeding stands. On the contrary, the accumulation of isopimaric acid, involved in defence (Kopper et al. 2005; López‐Goldar et al. 2020), was found to be higher in natural forests. The regulation of phenylalanine, which is involved in plant defence (Yadav and Chattopadhyay 2023), favoured the natural forests where it remained unaltered by drought, but it was downregulated in the breeding stands. Likewise, abietic acid and dehydroabietate, which are involved in defence against fungi and insects (Hamberger et al. 2011; Trapp and Croteau 2001), were upregulated in the natural forests compared to the breeding stands, which may be advantageous for the natural forests in terms of drought tolerance.

Carbohydrates are the general source of energy, and they are involved in growth, stress tolerance, and in maintaining osmotic balance under drought stress (Hunziker et al. 2024; Lewis and Smith 1967; Liao et al. 2022; Mathisson et al. 2021; Wu et al. 2023). Except for arabinose, which was upregulated in the natural forests compared to the breeding stands, other carbohydrates detected in the analysis were in favor of the breeding stands (raffinose, raffinose 2 and erythritol) as they were upregulated in breeding stands while their regulation was detected to be non‐significant in the natural forests under drought conditions. Likewise, downregulation of glutamic acid, an amino acid involved in growth and development (Liao et al. 2022), was relatively lower in the natural forests than in the breeding stands under drought conditions.

Succinic acid and oxoglutaric acid are the intermediates of the TCA cycle representing the energy‐yielding metabolism (Kiliç 2023; Zhang and Fernie 2018). Downregulation of oxoglutaric acid was found to be relatively lower in natural forests compared to the breeding stands under drought, suggesting that the breeding stands are more drought tolerant. However, drought did not affect the regulation of succinic acid in natural forests, which supports that the natural forests are more drought tolerant than breeding stands, as succinic acid was downregulated under drought in the breeding stands.

### 4.6 Metabolites Related to Hormone Analysis That Help in Alleviating Drought Stress

The regulation of metabolites with reference to hormone analysis is also somewhat in favor of the breeding stands, conferring better drought tolerance in them as compared to the natural Scots pine population (Table 2). In particular, the increase in ABA along with the increase in auxin conjugates in the breeding stands has been reported to alleviate drought responses by earlier studies. There was also an increase in IAA‐glucose and 2‐oxindole‐3‐acetic acid in the breeding stands, while the regulation of IAA‐aspartate remained non‐significant. ABA regulates plant growth and development along with the regulation of stress responses. ABA accumulates under drought stress and inhibits cell division, thereby inhibiting plant growth, which can help plants to survive better under drought conditions. It also helps in stomatal closure to prevent the loss of water during drought periods (Chen et al. 2020; Takahashi et al. 2020). The increase in auxin conjugates correlates well with drought/stress tolerance in plants, which has been demonstrated previously (Ludwig‐Müller 2011). In addition, there is an increase in tryptophan in the breeding stands, which is the precursor for auxin biosynthesis in plants (Mano and Nemoto 2012), where auxin regulates drought responses in plants particularly through signal transduction mediated by the interaction between auxin and ABA (Sharma et al. 2023).

Cytokinins were found to be increased in the breeding stands as compared to the natural forests, which may contribute to the intensification of the drought response in the breeding stands. In general, cytokinins are involved in cell division and growth. Cytokinins enhance water loss as they promote shoot growth, but they inhibit root growth, thereby limiting water uptake (Werner et al. 2001, 2010). Moreover, cytokinin suppresses the expression of ABA‐inducible genes leading to repression of drought stress tolerance (Tran et al. 2007). However, cytokinin also plays a protective role under drought stress as reviewed by Cortleven et al. (2019). For example, the application of cytokinins along with ABA alleviated drought stress in wheat, improving grain yield and biomass (Khosravi‐nejad et al. 2022). Thus, cytokinins affect the drought stress both negatively and positively, however, the increase/decrease in the cytokinin levels depend on the duration and severity of the drought (Iqbal et al. 2022).

12‐oxo‐Phytodienoic acid (OPDA) is a precursor of jasmonic acid, and jasmonic acid plays a central role in regulating plant defences (Ali and Baek 2020). OPDA controls growth processes in plants, as well as being involved in regulating jasmonate‐responsive genes that modulate defence responses (Liu and Park 2021). Salicylic acid increases plant tolerance to drought stress (Khan et al. 2015). Although the level of jasmonic acid remained unaltered in both populations, both salicylic acid and OPDA were detected to be decreased in the breeding stands, while their levels remained unchanged in the natural forests under drought conditions. The decrease in salicylic acid and OPDA can negatively govern drought tolerance in the breeding stands.

### 4.7 Association Between Variation in Metabolites and Distinct Canopy/Root Traits

Roles played by the metabolites detected in this study can be broadly classified into stress response/tolerance, defence, and growth; of these, growth is reflected in the phenotypic traits; for example, Canopy Height and Root Surface Area that are comparable to growth and biomass. The seedlings from breeding stands showed better growth under drought conditions as compared to the natural forests, which is evident from the phenotypic traits observed regarding the canopy (e.g., Canopy Height) and root development (e.g., Root Surface Area). Differences in these phenotypic characters between the breeding stands and natural forests can be correlated with variation in the metabolites. For instance, under drought conditions, carbohydrates involved in overall growth for example, raffinose (Hunziker et al. 2024; Wu et al. 2023) were found to be increased in the breeding stands along with higher Canopy Height compared to the natural forests, whereas the increase in raffinose was not statistically significant in the case of natural forests. Similar results were observed from the hormone analysis. For example, tryptophan involved in auxin biosynthesis (Mano and Nemoto 2012) and auxin conjugates were enhanced under drought coupled with higher Root Surface Area in breeding stands compared to the natural forests. Auxin is involved in the growth and development of aerial parts of the plants as well as roots (Ludwig‐Müller 2011). Auxin is mostly synthesised in shoots and is actively transported to roots where it promotes root development (Puig et al. 2012).

### 4.8 Metabolites Detected With Similar Fold Changes in Response to Drought Within Natural Forests and Breeding Stands

Several metabolites were detected with similar fold change differences caused by drought in the natural forests and breeding stands; however, fewer showed a fold change of more than double in the control versus drought conditions. Flavonoids like catechin, dihydromyricetin, epigallocatechin and kaempferol were found to be upregulated by drought that play a role in either stress tolerance in general or particularly drought tolerance and mediate the defence response (Likic et al. 2014; Witzell and Martín 2008; Yadav and Chattopadhyay 2023). A few examples of metabolites involved in stress tolerance found with decreased levels in both populations under drought conditions were ethanolamine, reported to enhance seedling tolerance to saline stress; glyceric acid, which induces tolerance to water stress (Li et al. 2019) and glycerol, which alleviates stress effects (Shen et al. 1999). Some metabolites involved in defence were found to be upregulated, while a few others were downregulated in response to drought. Dodecanoic acid, having antibacterial properties and known to improve drought resistance (Medeiros et al. 2015; Zhang, Du et al. 2022) and gallic acid, involved in the acclimation of plants to drought stress (Zhang, Ran et al. 2022) were among the defence related metabolites that were upregulated under drought in both populations. Particularly in conifers, dodecanoic acid was detected in response to drought in *Pinus taeda* needles (Wu et al. 2023). However, amino acids like beta‐alanine (Parthasarathy et al. 2019) and threonic acid (Wen et al. 2023) involved in defence, were downregulated in both populations under drought stress.

The accumulation of soluble sugars under drought plays a central role in maintaining osmotic balance, which helps in the regulation of gene expression and signaling (Kumar et al. 2021; Rosa et al. 2009), while also supporting overall growth and development (as a source of energy) and stress tolerance. Elevated levels of sugars such as fucose, isomaltose, maltose, glucose and sucrose were found under drought in both populations. Glucose and sucrose were earlier reported in pine species (de Simón et al. 2017) in the context of drought response. TCA‐related metabolites such as fumaric acid and malic acid, which are considered to be part of energy‐yielding metabolism (Zhang and Fernie 2018) were found to be decreased in both populations under drought conditions.

## 5 Conclusions

This is the first study that reports the effects of breeding on the drought‐tolerant capacity of Scots pine forests in Sweden, a species of significant economic importance to European forestry. The analysis, combining high‐throughput image‐based phenotyping with metabolomic profiling and hormone analysis, suggests that breeding stands may be more drought tolerant compared to related natural forests from which those trees in the breeding stands were selected through one breeding cycle. However, these conclusions are based on young Scots pine seedlings grown under controlled greenhouse conditions, and further validation in field settings is required. Despite this, the methodology employed in this work to assess drought tolerance and plant architecture could prove valuable for seedling selection in nurseries, enhancing drought resilience and supporting further research on forest management focused on climate change adaptability.

## Conflicts of Interest

The authors declare no conflicts of interest.

## Supporting information


**Figure S1.** Sites from where the populations were sampled. Blue dots represent breeding stands and green dots represent natural forests: Jokkmokk (Karatj‐Råvvåive 66°41′14.2″ N 18°56′37.4″ E), Arjeplog (66°18′15.8″ N 18°21′6.5″ E), and Jämtland (Källberget‐Storberget, 63°23′52.1″ N 15°28′0.6″ E). JN: Jokkmokk Natural, JS: Jokkmokk Breeding, AN: Arjeplog Natural, AS: Arjeplog Breeding, ON: Jämtland Natural, OS: Jämtland Breeding.
**Figure S2.** Water potential values of the soil during the experiment for both control (orange) and drought (blue) treatments. The arrow indicates the start of the drought treatment.
**Figure S3.** Correlogram between the measured variables for control conditions. Only significant (*p* < 0.05) correlations are shown. Coloured values represent Pearson r coefficient. Green line close to the variables name indicates variables corresponding to canopy phenotype meanwhile magenta line indicates variables corresponding to root phenotype.
**Figure S4.** Correlogram between the measured variables for drought conditions. Only significant (*p* < 0.05) correlations are shown. Coloured values represent Pearson r coefficient. Green line close to the variables name indicates variables corresponding to canopy phenotype meanwhile magenta line indicates variables corresponding to root phenotype.
**Figure S5.** Narrow‐sense heritability estimates (h2) of for breeding stands and natural forests tested in drought and controlled conditions.
**Figure S6.** The PCA with the multivariate analysis performed using SIMCA showing a clear separation between the control and drought samples, however, no separation was observed between the breeding stands and the natural forests with reference to (a) metabolite analysis and (b) hormone analysis.

**Table S1.** List of families included in the study. The name of the mother is always included, the father’s name is not included for open pollination families. Same names mean same fathers. AN: Arjeplog Natural, AS: Arjeplog Breeding, JN: Jokkmokk Natural, JS: Jokkmokk Breeding, OS: Jämtland Breeding.
**Table S2.** List of phenotypical traits analysed, organ and the type of distribution used in the GLM models: Family (a) or Source (b). The significance of each effect in the GLM model is shown. Each term of the model was checked with Analysis of Deviance (Type III) test. Level of significance is included as follows: ns, non‐significant; *, *p* < 0.05; **, *p* < 0.01; ***, *p* < 0.001.
**Table S3.** List of phenotypical traits analysed and treatment (Control or Drought) with their estimated coefficient of additive genetic variance (CVA%), narrow‐sense heritability values and standard error.
**Table S4.** List of metabolites detected with absolute fold change difference of less than 0.5 (log2) between the comparisons—control versus drought breeding stands and control versus drought natural forests.
**Table S5.** List of metabolites from hormone analysis detected with fold change difference of less than 0.5 (log2) between the comparisons—control versus drought breeding stands and control versus drought natural forests.

**Data S1.** Methods.

## Data Availability

Data used for the research described in the article is available in the article and the supplementary information.
