# Supplementary material for: Implications of Breeding for Growth on Drought Tolerance in Scots Pine ( Pinus sylvestris L.)—Insights From Metabolomics and High‐Throughput Plant Architecture Analysis
Source: Evol Appl. 2025 Jun 23;18(6):e70122. doi: 10.1111/eva.70122 (PMC12185384; doi:10.1111/eva.70122)

Figure S1 Sites from where the populations were sampled. Blue dots represent breeding stands and green dots represent natural forests: Jokkmokk (Karatj-Råvvåive 66º41'14.2"N 18º56'37.4"E), Arjeplog (66º18'15.8"N 18º21'6.5"E), and Jämtland (Källberget-Storberget, 63º23'52.1"N 15º28'0.6"E).
JN: Jokkmokk Natural, JS: Jokkmokk Breeding, AN: Arjeplog Natural, AS: Arjeplog Breeding, ON: Jämtland Natural, OS: Jämtland Breeding

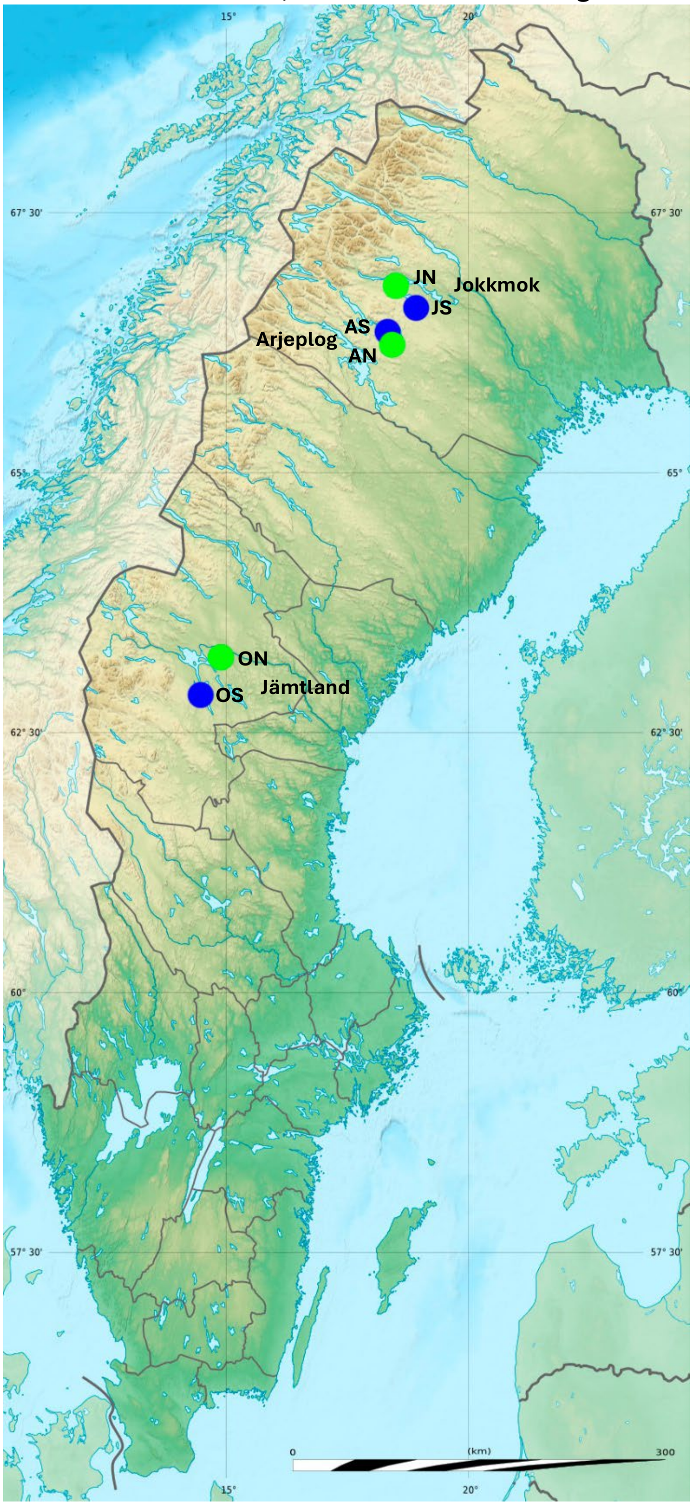

Figure S2 Water potential values of the soil during the experiment for both control (orange) and drought (blue) treatments. The arrow indicates the start of the drought treatment.

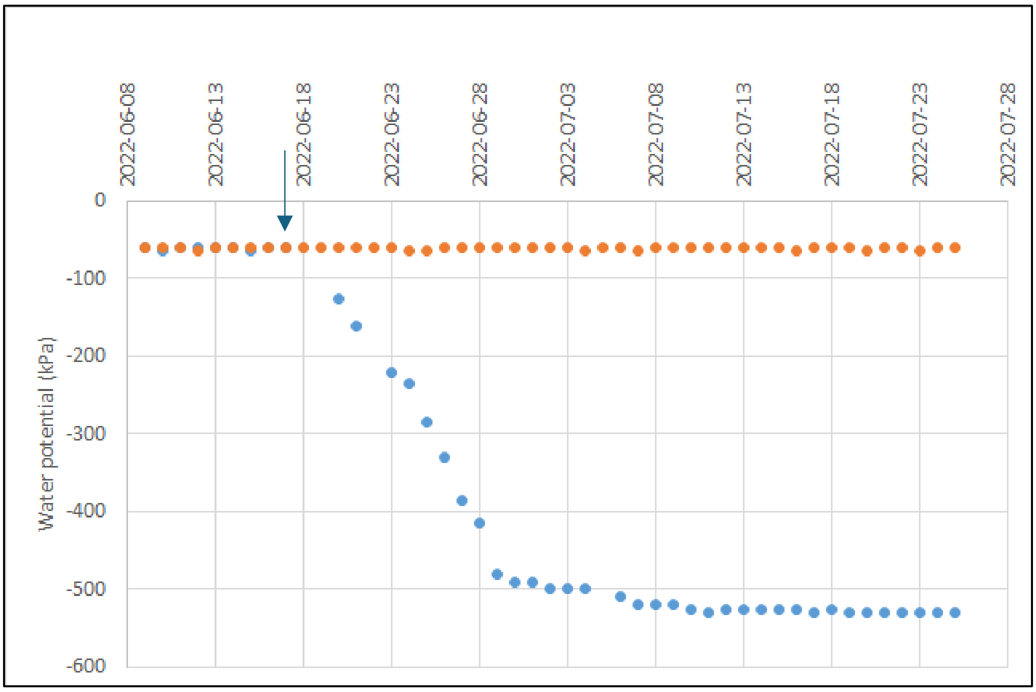

Figure S3. Correlogram between the measured variables for control conditions. Only significant ($p<0.05$) correlations are shown. Coloured values represent Pearson r coefficient. Green line close to the variables name indicates variables corresponding to canopy phenotype meanwhile magenta line indicates variables corresponding to root phenotype.

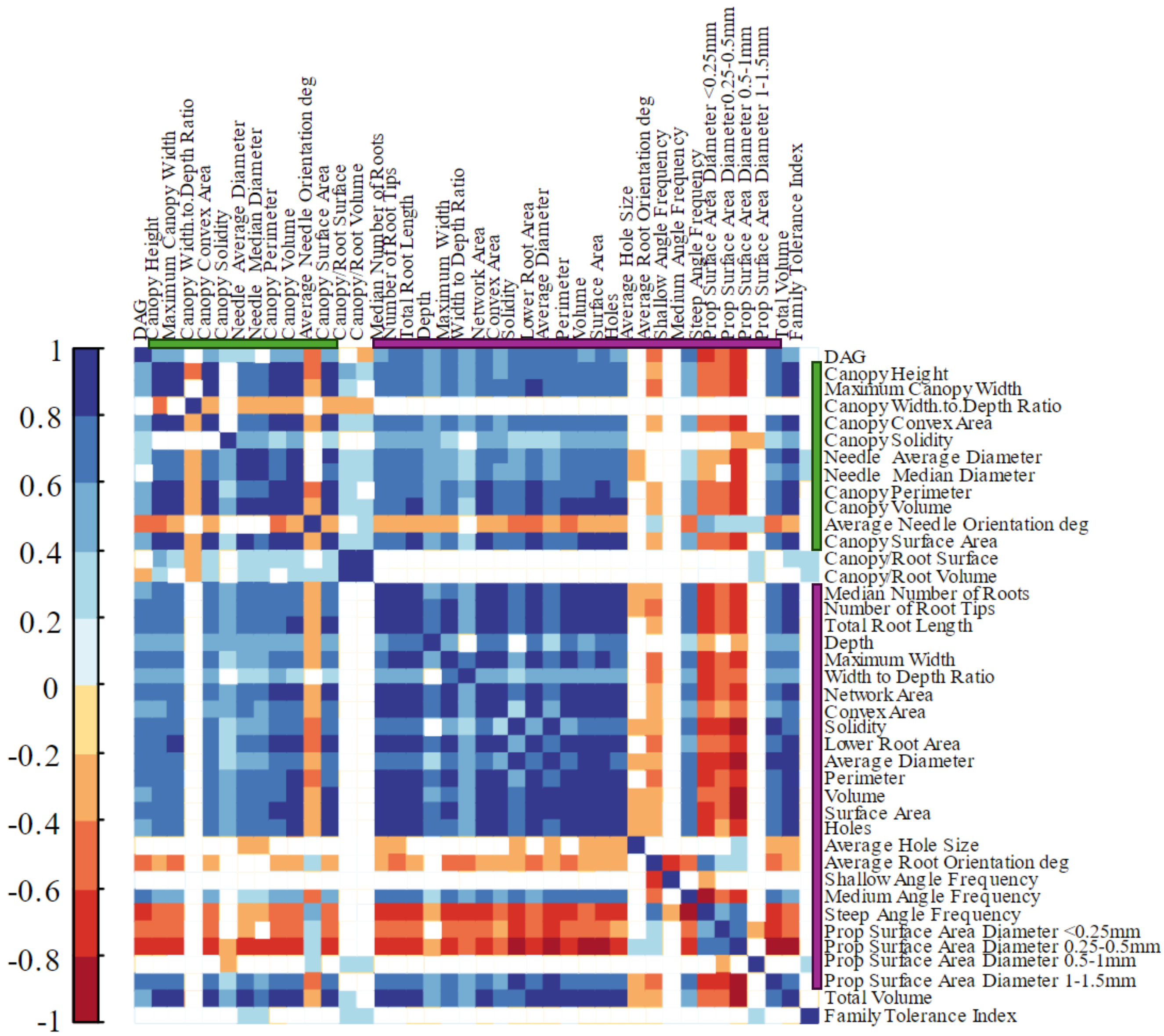

Figure S4 Correlogram between the measured variables for drought conditions. Only significant (p<0.05) correlations are shown. Coloured values represent Pearson r coefficient. Green line close to the variables name indicates variables corresponding to canopy phenotype meanwhile magenta line indicates variables corresponding to root phenotype.

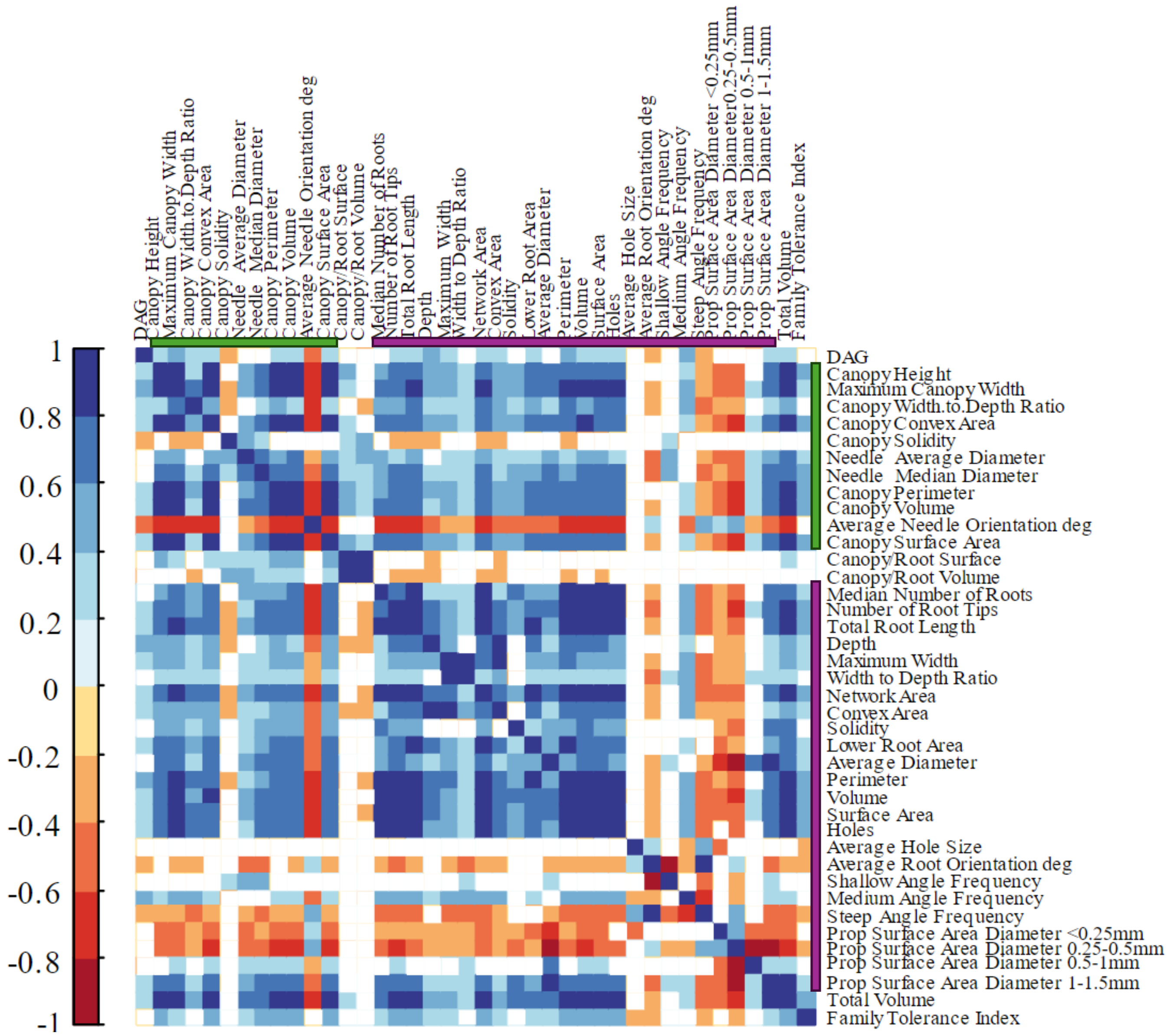

Figure S5 Narrow-sense heritability estimates (h2) of for breeding stands and natural forests tested in drought and controlled conditions.

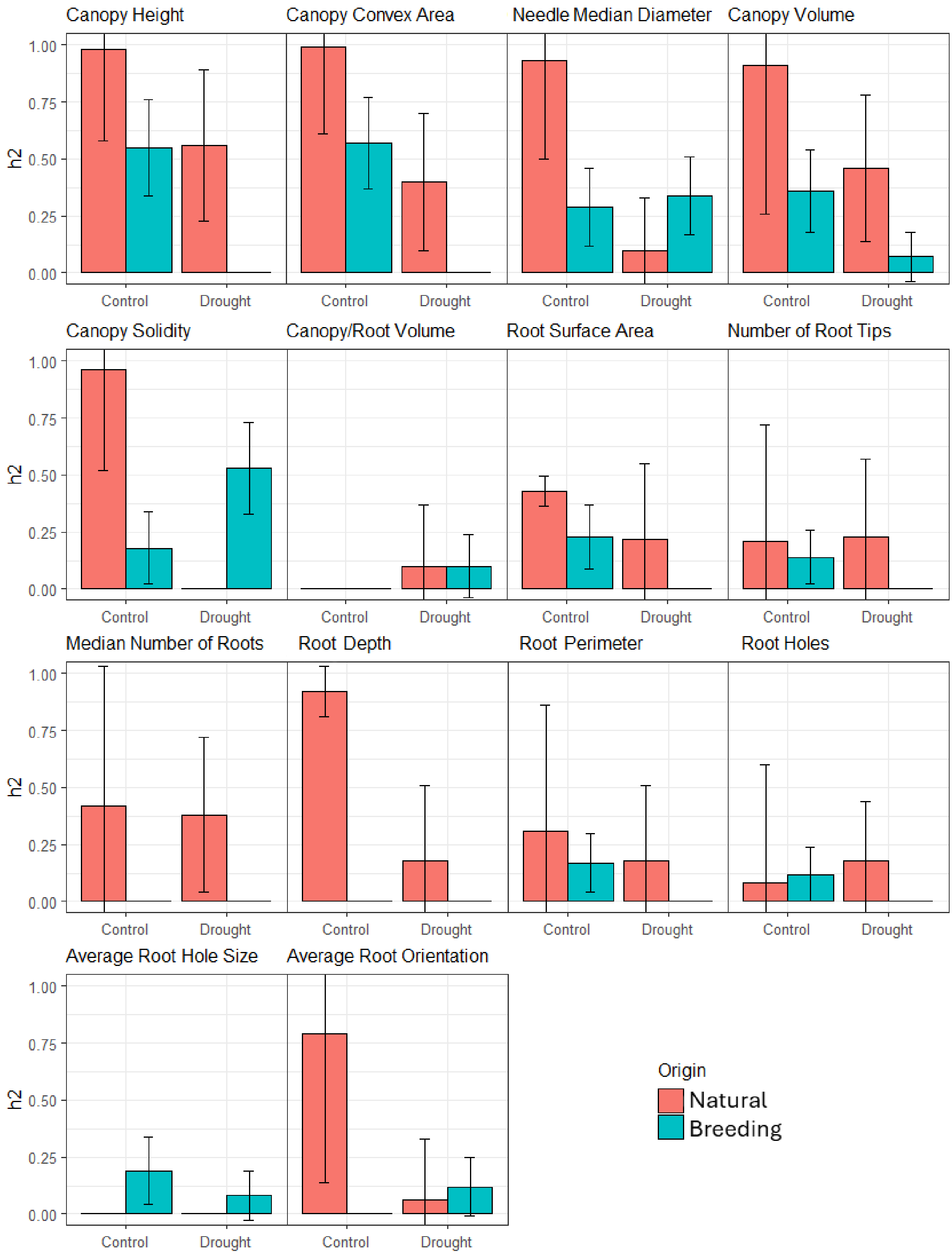

Figure S6 The PCA with the multivariate analysis performed using SIMCA showing a clear separation between the control and drought samples, however, no separation was observed between the breeding stands and the natural forests with reference to (a) metabolite analysis and (b) hormone analysis.

(a)

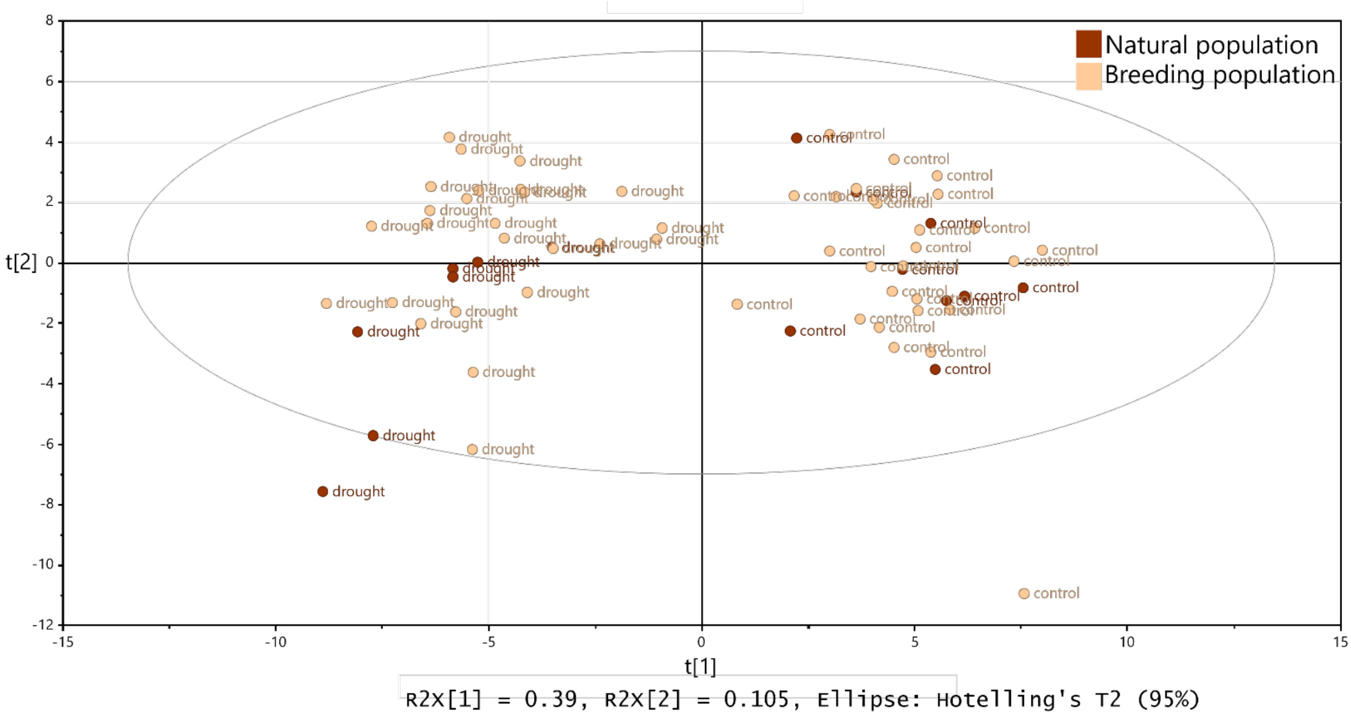

(b)

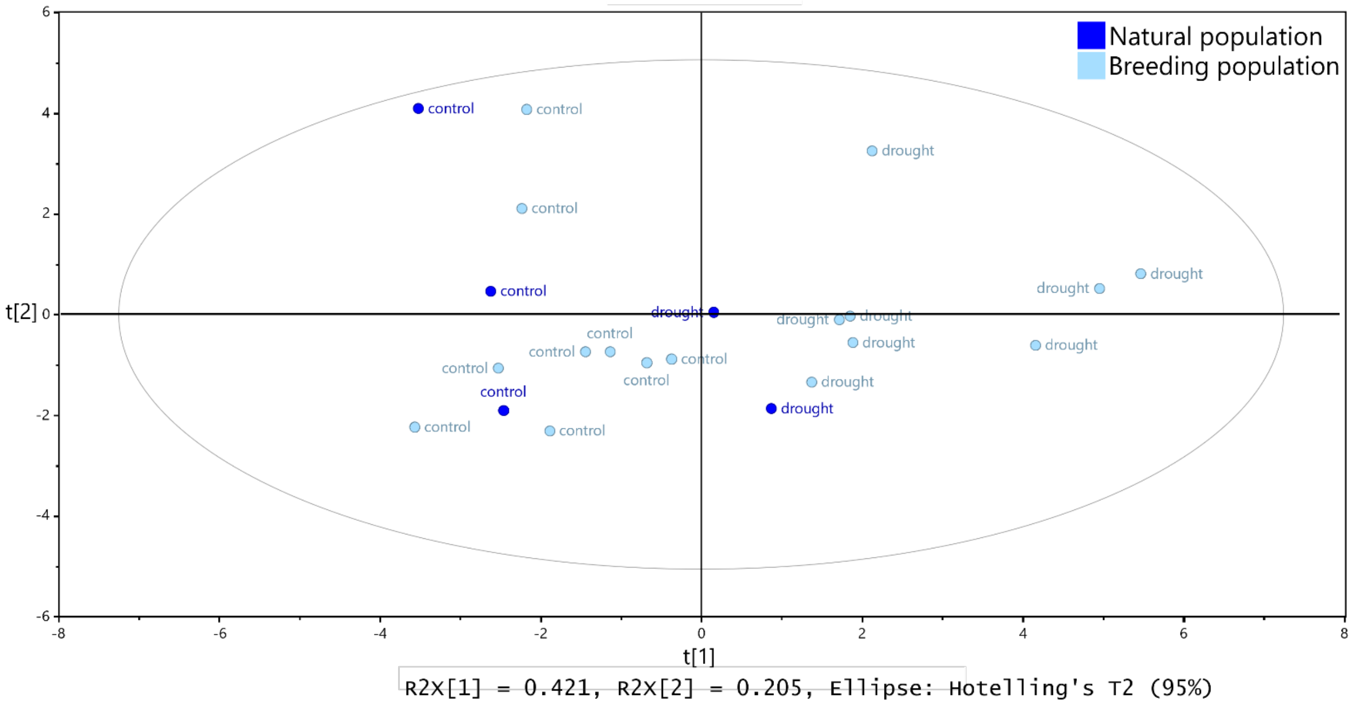

Supplement: Supplementary file 1 — Figure S1. Sites from where the populations were sampled. Blue dots represent breeding stands and green dots represent natural forests: Jokkmokk (Karatj‐Råvvåive 66°41′14.2″ N 18°56′37.4″ E), Arjeplog (66°18′15.8″ N 18°21′6.5″ E), and Jämtland (Källberget‐Storberget, 63°23′52.1″ N 15°28′0.6″ E). JN: Jokkmokk Natural, JS: Jokkmokk Breeding, AN: Arjeplog Natural, AS: Arjeplog Breeding, ON: Jämtland Natural, OS: Jämtland Breeding. Figure S2. Water potential values of the soil during the experiment for both control (orange) and drought (blue) treatments. The arrow indicates the start of the drought treatment. Figure S3. Correlogram between the measured variables for control conditions. Only significant (p < 0.05) correlations are shown. Coloured values represent Pearson r coefficient. Green line close to the variables name indicates variables corresponding to canopy phenotype meanwhile magenta line indicates variables corresponding to root phenotype. Figure S4. Correlogram between the measured variables for drought conditions. Only significant (p < 0.05) correlations are shown. Coloured values represent Pearson r coefficient. Green line close to the variables name indicates variables corresponding to canopy phenotype meanwhile magenta line indicates variables corresponding to root phenotype. Figure S5. Narrow‐sense heritability estimates (h2) of for breeding stands and natural forests tested in drought and controlled conditions. Figure S6. The PCA with the multivariate analysis performed using SIMCA showing a clear separation between the control and drought samples, however, no separation was observed between the breeding stands and the natural forests with reference to (a) metabolite analysis and (b) hormone analysis. [file EVA-18-e70122-s002.pdf]
