# Supplementary material for: Implications of Breeding for Growth on Drought Tolerance in Scots Pine ( Pinus sylvestris L.)—Insights From Metabolomics and High‐Throughput Plant Architecture Analysis
Source: Evol Appl. 2025 Jun 23;18(6):e70122. doi: 10.1111/eva.70122 (PMC12185384; doi:10.1111/eva.70122)
Supplement: Supplementary file 2 — Table S1. List of families included in the study. The name of the mother is always included, the father’s name is not included for open pollination families. Same names mean same fathers. AN: Arjeplog Natural, AS: Arjeplog Breeding, JN: Jokkmokk Natural, JS: Jokkmokk Breeding, OS: Jämtland Breeding. Table S2. List of phenotypical traits analysed, organ and the type of distribution used in the GLM models: Family (a) or Source (b). The significance of each effect in the GLM model is shown. Each term of the model was checked with Analysis of Deviance (Type III) test. Level of significance is included as follows: ns, non‐significant; *, p < 0.05; **, p < 0.01; ***, p < 0.001. Table S3. List of phenotypical traits analysed and treatment (Control or Drought) with their estimated coefficient of additive genetic variance (CVA%), narrow‐sense heritability values and standard error. Table S4. List of metabolites detected with absolute fold change difference of less than 0.5 (log2) between the comparisons—control versus drought breeding stands and control versus drought natural forests. Table S5. List of metabolites from hormone analysis detected with fold change difference of less than 0.5 (log2) between the comparisons—control versus drought breeding stands and control versus drought natural forests. [file EVA-18-e70122-s003.pdf]

Table S1 List of families included in the study. The name of the mother is always included, the father's name is not included for open pollination families. Same names mean same fathers.
AN: Arjeplog Natural, AS: Arjeplog Breeding, JN: Jokkmokk Natural, JS: Jokkmokk Breeding, OS: Jämtland Breeding.

Family	MotherID	FatherID	Origin	Location	Number Control	Number Drought
AN 5	ANM5	Open pollination	Natural	Arjeplog	5	3
AN 10	ANM10	Open pollination	Natural	Arjeplog	4	3
AN 16	ANM16	Open pollination	Natural	Arjeplog	4	7
AN 18	ANM18	Open pollination	Natural	Arjeplog	5	7
AS 1	AM1	Open pollination	Breeding	Arjeplog	3	6
AS 2	AM2	Open pollination	Breeding	Arjeplog	5	8
AS 4	AM3	Open pollination	Breeding	Arjeplog	3	8
AS 6	AM4	Open pollination	Breeding	Arjeplog	3	9
AS 7	AM4	Open pollination	Breeding	Arjeplog	5	6
AS 8	AM5	Open pollination	Breeding	Arjeplog	5	7
JN 1	JNM1	Open pollination	Natural	Jokkmokk	4	9
JN 2	JNM2	Open pollination	Natural	Jokkmokk	3	4
JN 4	JNM4	Open pollination	Natural	Jokkmokk	4	6
JN 5	JNM5	Open pollination	Natural	Jokkmokk	5	9
JN 13	JNM13	Open pollination	Natural	Jokkmokk	3	4
JN 15	JNM15	Open pollination	Natural	Jokkmokk	4	6
JN 18	JNM18	Open pollination	Natural	Jokkmokk	5	9
JS 11	JM5	F1	Breeding	Jokkmokk	6	6
OS 1	OM1	F3	Breeding	Jämtland	8	9
OS 2	OM2	Open pollination	Breeding	Jämtland	6	6
OS 3	OM3	F3	Breeding	Jämtland	11	9
OS 4	OM4	F3	Breeding	Jämtland	7	5
OS 5	OM4	F8	Breeding	Jämtland	9	6
OS 6	OM5	F9	Breeding	Jämtland	9	8
OS 7	OM6	F3	Breeding	Jämtland	11	12
OS 8	OM7	F10	Breeding	Jämtland	9	8
OS 9	OM8	F9	Breeding	Jämtland	9	10
OS 10	OM9	F4	Breeding	Jämtland	10	10
OS 11	OM10	F3	Breeding	Jämtland	6	7
OS 12	OM9	F3	Breeding	Jämtland	6	6
OS 14	OM8	F3	Breeding	Jämtland	8	7
OS 15	OM11	F3	Breeding	Jämtland	10	12
OS 16	OM3	F5	Breeding	Jämtland	10	11
OS 17	OM12	F6	Breeding	Jämtland	7	7
OS 18	OM13	F7	Breeding	Jämtland	7	8

Table S2 List of phenotypical variables analysed, organ and the type of distribution used in the GLM models: Family (a) or Population (b). The significance of each effect in the GLM model is shown. Each term of the model was checked with Analysis of Deviance (Type III) test
Level of significance is included as follows: n.s: non-significant, *: p <0.05, **: p<0.01, ***: p<0.001.

					Pr(>Chisq)		
**a) (Family Model) Variable**	**Organ**	**Model**	**Family**	**Treatment**	**Family:Treatment**	**DAG**	**Block**
Canopy Height	Canopy	Gamma	***	*	n.s.	***	n.s.
Maximum Canopy Width	Canopy	Gaussian	***	**	n.s.	***	n.s.
Canopy Width to Depth Ratio	Canopy	Gaussian	n.s.	n.s.	*	*	*
Canopy Convex Area	Canopy	Gamma	***	***	n.s.	***	n.s.
Canopy Solidity	Canopy	Gamma	**	n.s.	*	***	***
Needle Average Diameter	Canopy	Gaussian	***	n.s.	n.s.	***	**
Needle Median Diameter	Canopy	Gamma	***	n.s.	n.s.	***	***
Canopy Perimeter	Canopy	Gamma	***	**	n.s.	***	**
Canopy Volume	Canopy	Gamma	***	n.s.	n.s.	***	n.s.
Average Needle Orientation	Canopy	Gaussian	n.s.	**	*	***	n.s.
Canopy Surface Area	Canopy	Gamma	***	*	n.s.	***	**
Canopy/Root Surface		Gaussian	*	n.s.	n.s.	**	n.s.
Canopy/Root Volume		Gaussian	n.s.	n.s.	n.s.	**	*
Median Number of Roots	Root	Gamma	n.s.	n.s.	n.s.	***	***
Number of Root Tips	Root	Gamma	*	n.s.	n.s.	***	n.s.
Total Root Length	Root	Gamma	**	n.s.	n.s.	***	**
Depth	Root	Gaussian	n.s.	n.s.	n.s.	***	n.s.
Width to Depth Ratio	Root	Gamma	n.s.	n.s.	n.s.	n.s.	**
Network Area	Root	Gamma	**	n.s.	n.s.	***	*
Convex Area	Root	Gamma	n.s.	n.s.	n.s.	***	***
Solidity	Root	Gamma	n.s.	n.s.	n.s.	***	*
Lower Root Area	Root	Gamma	n.s.	n.s.	n.s.	***	**
Average Diameter	Root	Gamma	**	n.s.	n.s.	***	***
Perimeter	Root	Gamma	*	n.s.	n.s.	***	***
Volume	Root	Gamma	***	n.s.	n.s.	***	n.s.
Surface Area	Root	Gamma	***	n.s.	n.s.	***	n.s.
Holes	Root	Gamma	**	n.s.	n.s.	***	***
Average Hole Size	Root	Gamma	n.s.	n.s.	n.s.	n.s.	n.s.
Average Root Orientation	Root	Gamma	n.s.	n.s.	n.s.	***	**
Shallow Angle Frequency	Root	Gamma	n.s.	n.s.	n.s.	n.s.	**
Medium Angle Frequency	Root	Gamma	n.s.	*	n.s.	***	n.s.
Steep Angle Frequency	Root	Gamma	n.s.	n.s.	n.s.	***	**
Prop Surface Area Diameter Range 1	Root	Gamma	*	n.s.	n.s.	***	*
Prop Surface Area Diameter Range 2	Root	Gamma	**	n.s.	n.s.	***	n.s.
Prop Surface Area Diameter Range 3	Root	Gamma	n.s.	n.s.	n.s.	***	***
Prop Surface Area Diameter Range 4	Root	Gamma	n.s.	n.s.	n.s.	***	***

					Pr(>Chisq)		
**b) (Origin Model) Variable**	**Organ**	**Model**	**Population**	**Treatment**	**Population:Treatment**	**DAG**	**Block**
Canopy Height	Canopy	Gamma	***	***	n.s.	***	n.s.
Maximum Canopy Width	Canopy	Gaussian	***	***	n.s.	***	n.s.
Canopy Width to Depth Ratio	Canopy	Gaussian	**	n.s.	**	**	*
Canopy Convex Area	Canopy	Gamma	***	***	n.s.	***	n.s.
Canopy Solidity	Canopy	Gamma	n.s.	n.s.	n.s.	***	***
Needle Average Diameter	Canopy	Gaussian	***	n.s.	n.s.	***	**
Needle Median Diameter	Canopy	Gamma	***	n.s.	n.s.	***	***
Canopy Perimeter	Canopy	Gamma	***	***	n.s.	***	*
Canopy Volume	Canopy	Gamma	***	***	n.s.	***	n.s.
Average Needle Orientation	Canopy	Gaussian	n.s.	***	*	***	n.s.
Canopy Surface Area	Canopy	Gamma	***	***	n.s.	***	n.s.
Canopy/Root Surface		Gaussian	**	n.s.	n.s.	*	n.s.
Canopy/Root Volume		Gaussian	**	n.s.	*	*	*
Median Number of Roots	Root	Gamma	n.s.	***	n.s.	***	***
Number of Root Tips	Root	Gamma	*	***	n.s.	n.s.	n.s.
Total Root Length	Root	Gamma	n.s.	***	n.s.	***	***
Depth	Root	Gaussian	n.s.	***	n.s.	***	n.s.
Width to Depth Ratio	Root	Gamma	n.s.	***	n.s.	**	**
Network Area	Root	Gamma	*	***	n.s.	***	n.s.
Convex Area	Root	Gamma	n.s.	n.s.	n.s.	***	***
Solidity	Root	Gamma	n.s.	***	n.s.	***	**
Lower Root Area	Root	Gamma	n.s.	***	n.s.	***	**
Average Diameter	Root	Gamma	*	***	n.s.	***	***
Perimeter	Root	Gamma	n.s.	***	n.s.	***	***
Volume	Root	Gamma	*	***	n.s.	***	*
Surface Area	Root	Gamma	*	***	n.s.	***	n.s.
Holes	Root	Gamma	*	**	n.s.	***	***
Average Hole Size	Root	Gamma	n.s.	n.s.	n.s.	n.s.	n.s.
Average Root Orientation	Root	Gamma	n.s.	***	n.s.	***	**
Shallow Angle Frequency	Root	Gamma	n.s.	**	n.s.	*	**
Medium Angle Frequency	Root	Gamma	n.s.	n.s.	n.s.	***	n.s.
Steep Angle Frequency	Root	Gamma	n.s.	**	n.s.	***	**
Prop Surface Area Diameter Range 1	Root	Gamma	n.s.	**	n.s.	***	***
Prop Surface Area Diameter Range 2	Root	Gamma	*	***	n.s.	***	***
Prop Surface Area Diameter Range 3	Root	Gamma	n.s.	n.s.	n.s.	***	***
Prop Surface Area Diameter Range 4	Root	Gamma	n.s.	***	*	***	***

Table S3. List of phenotypical variables analysed and treatment (Control or Drought) with their estimated coefficient of additive genetic variance (CVA%), narrow-sense heritability values and standard error.

Traits	Breeding-Control		Natural-Control		Breeding-Drought		Natural-Drought	
	CVA%	h2	CVA%	h2	CVA%	h2	CVA%	h2
Canopy_Height_mm	11.28	0.55 (0.21)	15.14	0.98 (0.40)	0.01	0	15.14	0.56 (0.33)
Needle_Median_Diameter_mm	3.81	0.29 (0.17)	3.25	0.93 (0.43)	4.57	0.34 (0.17)	3.25	0.10 (0.23)
Root_Surface_Area_mm2	20.58	0.23 (0.14)	23.44	0.43 (0.64)	0.01	0	23.44	0.22 (0.33)
Average_Root_Hole_Size_mm2	23.96	0.19 (0.15)	0.07	0	20.75	0.08 (0.11)	0.07	0
Average_Root_Orientation_deg	0	0	1.18	0.79 (0.65)	1.4	0.12 (0.13)	1.18	0.06 (0.27)
Total_Canopy_Length_mm	20.77	0.46 (0.20)	36	0.99 (0.39)	0.02	0	35.7	0.43 (0.30)
Canopy_Network_Area_mm2	22.66	0.48 (0.20)	38.5	0.99 (0.39)	0.03	0	37	0.40 (0.30)
Canopy_Convex_Area_mm2	20.12	0.57 (0.20)	37.12	0.99 (0.38)	0.03	0	37.84	0.47 (0.32)
Canopy_Solidity	5.96	0.18 (0.16)	13.36	0.96 (0.44)	10.21	0.53 (0.20)	0.1	0
Canopy_Volume_mm3	28.63	0.36 (0.18)	47.8	0.91 (0.65)	12.98	0.07 (0.11)	41.53	0.46 (0.32)
Canopy/Root_Surface	0.04	0	0.03	0	8.33	0.04 (0.11)	5.15	0.013 (0.23)
Canopy/Root_Volume	0.01	0	0.04	0	14.43	0.10 (0.14)	15.83	0.10 (0.27)
Median_Number_of_Roots	0.03	0	19.76	0.42 (0.61)	0.01	0	29.93	0.38 (0.34)
Maximum_Number_of_Roots	8.56	0.14 (0.13)	12.6	0.26 (0.52)	0	0	0.03	0
Number_of_Root_Tips	14.26	0.14 (0.12)	17.41	0.21 (0.51)	0.03	0	21.46	0.23 (0.34)
Total_Root_Length_mm	15.64	0.20 (0.14)	21.07	0.36 (0.60)	0	0	23.44	0.24 (0.33)
Root_Depth_mm	0.01	0	18	0.92 (0.11)	0.01	0	8.19	0.18 (0.33)
Root_Perimeter_mm	13.15	0.17 (0.13)	18.58	0.31 (0.55)	0.01	0	0.59	0.18 (0.33)
Root_Holes	16.27	0.12 (0.12)	13.59	0.08 (0.52)	0.01	0	24.82	0.18 (0.26)

Table S4 List of metabolites detected with absolute fold change difference of less than 0.5 (log2) between the comparisons – control versus drought breeding stands and control versus drought natural forests.

	Control versus drought Breeding stands		Control versus drought Natural forests					
**Metabolite**	**Fold change under drought (log2)**	**P-value**	**Fold change under drought (log2)**	**P-value**	**Absolute value of fold change difference between breeding and natural population (log2)**	**Metabolite class**	**Function**	**Reference**
alpha-Linolenic acid	-0.19	0.03	--	Not significant	0.2	Fatty acids	Stress response - Mediates drought responses	(Zi, Zhou, & Wu, 2022)
D-Pinitol	0.55	0.00	0.58	0.00	0.0	Alcohols and polyols	Stress response and cellular signalling, growth regulators	(Guo & Oosterhuis, 1997)
myo-Inositol	-0.41	0.00	-0.47	0.00	0.1	Alcohols and polyols	Stress response and cellular signalling, growth regulators	(Kumar, Patel, Kumar, Bajpai, & Siddique, 2021; Valluru & Van den Ende, 2011)
scyllo-Inositol	0.37	0.00	0.72	0.00	0.3	Alcohols and polyols	Stress response and cellular signalling, growth regulators	(Valluru & Van den Ende, 2011)
Valine	-0.49	0.01	-0.76	0.03	0.3	Amino acids and peptides	Stress response, reduces vegetative growth under drought	(Charlton et al., 2008; Li et al., 2020)
beta-Sitosterol	--	Not significant	0.33	0.02	0.3	Sterols	Stress tolerance, increased levels detected under drought	(de Simón et al., 2017; Shahzad et al., 2021)
beta-Sitosterol 2	--	Not significant	0.33	0.02	0.3	Sterols	Stress tolerance, increased levels detected under drought	(de Simón et al., 2017; Shahzad et al., 2021)
Putrescine	-0.45	0.00	-0.52	0.04	0.1	Fatty amines	Stress tolerance - alleviates drought stress	(X. Y. Wang, Li, Wang, Li, & Dong, 2022)
Glycerol	-1.14	0.00	-0.93	0.00	0.2	Alcohols and polyols	Stress tolerance - alleviating stress effects	(X. X. Wang et al., 2019)
Catechin	2.67	0.00	2.27	0.00	0.4	Flavonoids	Stress tolerance, protection against biotic and abiotic stresses, cell growth	(Witzell & Martín, 2008)

Dihydromyricetin	3.99	0.00	3.56	0.00	0.4	Flavonoids	Stress tolerance/defense in general, role not explored in drought tolerance	(Yadav & Chattopadhyay, 2023)
Epigallocatechin	1.92	0.00	2.04	0.00	0.1	Flavonoids	Stress tolerance/defense, plays role in drought tolerance	(Yadav & Chattopadhyay, 2023)
Kaempferol	1.07	0.00	1.00	0.05	0.1	Flavonoids	Stress tolerance/defense, plays role in drought tolerance	(Likic, Sola, Ludwig-Müller, & Rusak, 2014)
Ethanolamine	-1.18	0.00	-1.03	0.00	0.2	Amines	Stress-tolerance	(Kogan, Kristoff, Benavides, & Tomaro, 2000)
Dehydroascorbic acid (DHAA)	0.35	0.00	0.37	0.01	0.0	Lactones	Stress-tolerance - cellular homeostasis	(Deutsch, 2000; Dreyer, 2021)
Glyceric acid	-0.89	0.00	-0.74	0.01	0.2	Carbohydrates Monosaccharides	Stress-tolerance - induces stress tolerance	(X. X. Wang et al., 2019)
Fructose	0.34	0.00	0.26	0.01	0.1	Carbohydrates Monosaccharides	Stress-tolerance, maintains osmotic balance under drought stress, energy and carbon source.	(Kumar et al., 2021; Rosa et al., 2009)
Fucose	1.11	0.00	1.19	0.00	0.1	Carbohydrates Monosaccharides	Stress-tolerance, maintains osmotic balance under drought stress, energy and carbon source.	(Kumar et al., 2021; Rosa et al., 2009)
Isomaltose	3.64	0.00	3.94	0.02	0.3	Carbohydrates Disaccharides	Stress-tolerance, maintains osmotic balance under drought stress, energy and carbon source.	(Kumar et al., 2021; Rosa et al., 2009)
Maltose	0.85	0.00	1.07	0.00	0.2	Carbohydrates Disaccharides	Stress-tolerance, maintains osmotic balance under drought stress, energy and carbon source.	(Kumar et al., 2021; Rosa et al., 2009)
Melibiose	3.64	0.00	3.94	0.02	0.3	Carbohydrates Disaccharides	Stress-tolerance, maintains osmotic balance under drought stress, energy and carbon source.	(Kumar et al., 2021; Rosa et al., 2009)
beta-Alanine	-1.87	0.00	-1.85	0.01	0.0	Amino acids and peptides	Defense	(Parthasarathy, Savka, & Hudson, 2019)
Threonic acid	-1.84	0.00	-1.80	0.00	0.0	Carbohydrates Monosaccharides	Defense	(Wen et al., 2023)
Dodecanoic acid or Lauric Acid	0.51	0.00	0.60	0.00	0.1	Fatty acids	Defense - antibacterial agent	(Medeiros et al., 2015; B. B. Zhang et al., 2022)
Ferulic acid	-0.12	0.04	--	Not significant	0.1	Phenylpropanoids	Defense - antioxidant, antimicrobial	(Kumar et al., 2021)
Serine	-1.32	0.00	-1.73	0.00	0.4	Amino acids and peptides	Defense - plant immunity	(X. M. Zhang et al., 2023)

Palmitic acid	-0.13	0.01	--	Not significant	0.1	Fatty acids	Defense - reduces disease incidences and promotes the growth	(Kumar et al., 2021)
Linoleic acid	-0.41	0.00	--	Not significant	0.4	Fatty acids	Defense against abiotic stress	(Kumar et al., 2021)
Gallic acid	0.78	0.00	0.68	0.01	0.1	Phenolic acids	Defense, acclimation of plant to drought stress	(X. Zhang et al., 2022)
Shikimic acid	0.18	0.00	--	Not significant	0.2	Phenolic acids	Lignin synthesis, defense	(Santos-Sánchez, Salas-Coronado, Hernández-Carlos, & Villanueva-Cañongo, 2019)
Pyroglutamic acid	-2.87	0.00	-3.20	0.00	0.3	Amino acids and peptides	Growth - combats productivity losses due to water scarcity	(Jiménez-Arias et al., 2019)
Glycine	-0.32	0.02	--	Not significant	0.3	Amino acids and peptides	Growth, stress resistance	(Ashraf & Foolad, 2007)
Glucose	0.52	0.00	0.45	0.00	0.1	Carbohydrates Monosaccharides	Growth & development	(Kumar et al., 2021; Rosa et al., 2009)
Sucrose	0.21	0.00	0.29	0.00	0.1	Carbohydrates Disaccharides	Growth & development	(Kumar et al., 2021; Rosa et al., 2009)
Aspartic acid	-1.72	0.00	-2.09	0.00	0.4	Amino acids and peptides	Growth and defense	(Kumar et al., 2021)
Levoglucosan	0.42	0.00	0.55	0.01	0.1	Oxepanes	Carbon source, growth	(de Simón et al., 2017)
Citramalic acid	-2.75	0.00	-2.66	0.02	0.1	Hydroxy acids	Rare metabolite in plants, may play role drought in conifers. Solubilizes soil phosphorus in P deficient soil	(Khorassani et al., 2011)
Fumaric acid	-1.21	0.00	-1.30	0.00	0.1	TCA acids	TCA - energy-yielding metabolism	(Y. J. Zhang & Fernie, 2018)
Malic acid	-1.42	0.00	-1.12	0.00	0.3	TCA acids	TCA - energy-yielding metabolism	(Y. J. Zhang & Fernie, 2018)

Table S5 List of metabolites from hormone analysis detected with fold change difference of less than 0.5 (log2) between the comparisons – control versus drought breeding stands and control versus drought natural forests.

		Control versus drought Breeding stands		Control versus drought Natural forests				
**Metabolite type**	**Metabolite**	**Fold change under drought (log2)**	**P-value**	**Fold change under drought (log2)**	**P-value**	**Absolute value of fold change difference between breeding & natural population (log2)**	**Function**	**Reference**
Cytokinin	cis-zeatin	0.49	0.01	0.19	0.01	0.3	Positive/negative effects on drought stress	(Cortleven et al., 2019; Tran et al., 2007)
Cytokinin	Trans-zeatin	0.50	0.01	0.21	0.02	0.3	Positive/negative effects on drought stress	(Cortleven et al., 2019; Tran et al., 2007)
Cytokinin	Trans-zeatin riboside	-0.36	0.04	--	Not significant	0.4	Positive/negative effects on drought stress	(Cortleven et al., 2019; Tran et al., 2007)

References

Ashraf, M., & Foolad, M. R. (2007). Roles of glycine betaine and proline in improving plant abiotic stress resistance. *Environmental and Experimental Botany, 59*(2), 206-216. doi:10.1016/j.envexpbot.2005.12.006

Charlton, A. J., Donarski, J. A., Harrison, M., Jones, S. A., Godward, J., Oehlschlager, S., . . . Domoney, C. (2008). Responses of the pea (Pisum sativum L.) leaf metabolome to drought stress assessed by nuclear magnetic resonance spectroscopy. *Metabolomics, 4*(4), 312-327. doi:10.1007/s11306-008-0128-0

Cortleven, A., Leuendorf, J. E., Frank, M., Pezzetta, D., Bolt, S., & Schmülling, T. (2019). Cytokinin action in response to abiotic and biotic stresses in plants. *Plant Cell and Environment, 42*(3), 998-1018. doi:10.1111/pce.13494

de Simón, B. F., Sanz, M., Cervera, M. T., Pinto, E., Aranda, I., & Cadahía, E. (2017). Leaf metabolic response to water deficit in Pinus pinaster Ait. relies upon ontogeny and genotype. *Environmental and Experimental Botany, 140*, 41-55. doi:10.1016/j.envexpbot.2017.05.017

Deutsch, J. C. (2000). Dehydroascorbic acid. *Journal of Chromatography A, 881*(1-2), 299-307. doi:Doi 10.1016/S0021-9673(00)00166-7

Dreyer, I. (2021). Nutrient cycling is an important mechanism for homeostasis in plant cells. *Plant Physiology, 187*(4), 2246-2261. doi:10.1093/plphys/kiab217

Guo, C. X., & Oosterhuis, D. M. (1997). Effect of water-deficit stress and genotypes on pinitol occurrence in soybean plants. *Environmental and Experimental Botany, 37*(2-3), 147-152. doi:Doi 10.1016/S0098-8472(96)01047-7

Jiménez-Arias, D., García-Machado, F. J., Morales-Sierra, S., Luis, J. C., Suarez, E., Hernández, M., . . . Borges, A. A. (2019). Lettuce plants treated with L-pyroglutamic acid increase yield under water deficit stress. *Environmental and Experimental Botany, 158*, 215-222. doi:10.1016/j.envexpbot.2018.10.034

Khorassani, R., Hettwer, U., Ratzinger, A., Steingrobe, B., Karlovsky, P., & Claassen, N. (2011). Citramalic acid and salicylic acid in sugar beet root exudates solubilize soil phosphorus. *Bmc Plant Biology, 11*. doi:10.1186/1471-2229-11-121

Kogan, M. J., Kristoff, G., Benavides, M. P., & Tomaro, M. L. (2000). Effect of pre-treatment with ethanolamine on the response of Helianthus annuus L. to salt stress. *Plant Growth Regulation, 30*(1), 87-94. doi:Doi 10.1023/A:1006360912089

Kumar, M., Patel, M. K., Kumar, N., Bajpai, A. B., & Siddique, K. H. M. (2021). Metabolomics and Molecular Approaches Reveal Drought Stress Tolerance in Plants. *International Journal of Molecular Sciences, 22*(17). doi:10.3390/ijms22179108

Li, S. H., Peng, F. T., Xiao, Y. S., Gong, Q. T., Bao, Z. Y., Li, Y. Y., & Wu, X. L. (2020). Mechanisms of High Concentration Valine-Mediated Inhibition of Peach Tree Shoot Growth. *Frontiers in Plant Science, 11*. doi:10.3389/fpls.2020.603067

Likic, S., Sola, I., Ludwig-Müller, J., & Rusak, G. (2014). Involvement of kaempferol in the defence response of virus infected. *European Journal of Plant Pathology, 138*(2), 257-271. doi:10.1007/s10658-013-0326-0

Medeiros, M. J., Oliveira, D. S., Oliveira, M. T., Willadino, L., Houllou, L., & Santos, M. G. (2015). Ecophysiological, anatomical and biochemical aspects of in vitro culture of zygotic Syagrus coronata embryos and of young plants under drought stress. *Trees-Structure and Function, 29*(4), 1219-1233. doi:10.1007/s00468-015-1202-7

Parthasarathy, A., Savka, M. A., & Hudson, A. O. (2019). The Synthesis and Role of β-Alanine in Plants. *Frontiers in Plant Science, 10*. doi:10.3389/fpls.2019.00921

Rosa, M., Prado, C., Podazza, G., Interdonato, R., González, J. A., Hilal, M., & Prado, F. E. (2009). Soluble sugars-Metabolism, sensing and abiotic stress A complex network in the life of plants. *Plant Signaling & Behavior, 4*(5), 388-393. doi:DOI 10.4161/psb.4.5.8294

Santos-Sánchez, N. F., Salas-Coronado, R., Hernández-Carlos, B., & Villanueva-Cañongo, C. (2019). Shikimic acid pathway in biosynthesis of phenolic compounds. In *Plant Physiological Aspects of Phenolic Compounds*. London, UK: IntechOpen.

Shahzad, R., Ewas, M., Harlina, P. W., Khan, S. U., Zhenyuan, P., Nie, X. H., & Nishawy, E. (2021). β-Sitosterol differentially regulates key metabolites for growth improvement and stress tolerance in rice plants during prolonged UV-B stress. *Journal of Genetic Engineering and Biotechnology, 19*(1). doi:10.1186/s43141-021-00183-6

Tran, L. S. P., Urao, T., Qin, F., Maruyama, K., Kakimoto, T., Shinozaki, K., & Yamaguchi-Shinozaki, K. (2007). Functional analysis of AHK1/ATHK1 and cytokinin receptor histidine kinases in response to abscisic acid, drought, and salt stress in. *Proceedings of the National Academy of Sciences of the United States of America, 104*(51), 20623-20628. doi:10.1073/pnas.0706547105

Valluru, R., & Van den Ende, W. (2011). Myo-inositol and beyond - Emerging networks under stress. *Plant Science, 181*(4), 387-400. doi:10.1016/j.plantsci.2011.07.009

Wang, X. X., Guo, R., Li, M. X., Liu, Y., Zhao, M. L., Fu, H., . . . Shi, L. X. (2019). Metabolomics reveals the drought-tolerance mechanism in wild soybean (Glycine soja). *Acta Physiologiae Plantarum, 41*(9). doi:10.1007/s11738-019-2939-1

Wang, X. Y., Li, Y. P., Wang, X. J., Li, X. M., & Dong, S. K. (2022). Physiology and metabonomics reveal differences in drought resistance among soybean varieties. *Botanical Studies, 63*(1). doi:10.1186/s40529-022-00339-8

Wen, T., Ding, Z. X., Thomashow, L. S., Hale, L., Yang, S. D., Xie, P. H., . . . Yuan, J. (2023). Deciphering the mechanism of fungal pathogen-induced disease-suppressive soil. *New Phytologist, 238*(6), 2634-2650. doi:10.1111/nph.18886

Witzell, J., & Martín, J. A. (2008). Phenolic metabolites in the resistance of northern forest trees to pathogens - past experiences and future prospects. *Canadian Journal of Forest Research, 38*(11), 2711-2727. doi:10.1139/X08-112

Yadav, S., & Chattopadhyay, D. (2023). Lignin: the Building Block of Defense Responses to Stress in Plants. *Journal of Plant Growth Regulation, 42*(10), 6652-6666. doi:10.1007/s00344-023-10926-z

Zhang, B. B., Du, H., Sun, M. X., Wu, X. L., Li, Y. Y., Wang, Z., . . . Peng, F. T. (2022). Comparison of lauric acid and 12-hydroxylauric acid in the alleviation of drought stress in peach (Prunus persica (L.) Batsch). *Frontiers in Plant Science, 13*. doi:10.3389/fpls.2022.1025569

Zhang, X., Ran, W., Li, X. W., Zhang, J., Ye, M., Lin, S. B., . . . Sun, X. L. (2022). Exogenous Application of Gallic Acid Induces the Direct Defense of Tea Plant Against *Ectropis obliqua* Caterpillars. *Frontiers in Plant Science, 13*. doi:10.3389/fpls.2022.833489

Zhang, X. M., Tubergen, P. J., Agorsor, I. D. K., Khadka, P., Tembe, C., Denbow, C., . . . Danna, C. H. (2023). Elicitor-induced plant immunity relies on amino acids accumulation to delay the onset of bacterial virulence. *Plant Physiology, 192*(1), 601-615. doi:10.1093/plphys/kiad048

Zhang, Y. J., & Fernie, A. R. (2018). On the role of the tricarboxylic acid cycle in plant productivity. *Journal of Integrative Plant Biology, 60*(12), 1199-1216. doi:10.1111/jipb.12690

Zi, X., Zhou, S., & Wu, B. (2022). Alpha-Linolenic Acid Mediates Diverse Drought Responses in Maize (Zea mays L.) at Seedling and Flowering Stages. *Molecules, 27*(3). doi:10.3390/molecules27030771
