# Supplementary material for: Implications of Breeding for Growth on Drought Tolerance in Scots Pine ( Pinus sylvestris L.)—Insights From Metabolomics and High‐Throughput Plant Architecture Analysis
Source: Evol Appl. 2025 Jun 23;18(6):e70122. doi: 10.1111/eva.70122 (PMC12185384; doi:10.1111/eva.70122)
Supplement: Supplementary file 3 — Data S1. Methods. [file EVA-18-e70122-s001.pdf]

Gas chromatography–mass spectrometry (GC-MS) and hormone analysis

GC-MS and hormone analysis was performed with shoots of the seedlings, grounded and maintained under frozen conditions.

Sample Preparation: Sample preparation of 20 mg of grounded seedling sample was performed according to Gullberg et al (Gullberg et al. 2004). In detail, 1 mL of ice-cold 60% aqueous acetonitrile (v/v) including internal standards (L-proline-13C5, alpha-ketoglutarate-13C4, myristic acid-13C3, cholesterol-D7, succinic acid-D4, salicylic acid-D6, L-glutamic acid-13C5,15N, putrescine-D4, hexadecanoic acid-13C4, D-glucose-13C6, D-sucrose-13C12). The sample was shaken with one tungsten bead in a mixer mill at 30 Hz for 3 minutes, the bead was removed, and the sample was centrifuged at +4 °C, 14 000 rpm (18 620g), for 10 minutes. 50 µl of the supernatant was transferred to micro vials and evaporated to dryness in a speed-vac concentrator. Solvents were evaporated and the samples were stored at -80 °C until analysis. A small aliquot of the remaining supernatants was pooled and used to create quality control (QC) samples. The samples were analyzed in batches according to a randomized run order.

GC-MS Analysis: Derivatization and GC-MS analysis were performed as described previously (Gullberg et al. 2004). 1 µL of the derivatized sample was injected in splitless mode by a L-PAL3 autosampler (CTC Analytics AG, Switzerland) into an Agilent 7890B gas chromatograph equipped with a 10 m x 0.18 mm fused silica capillary column with a chemically bonded 0.18 µm Rxi-5 Sil MS stationary phase (Restek Corporation, U.S.) The injector temperature was 270 °C, the purge flow rate was 20 mL min-1 and the purge was turned on after 60 seconds. The gas flow rate through the column was 1 mL min-1, the column temperature was held at 70 °C for 2 minutes, then increased by 40 °C min-1 to 320 °C, and held there for 2 minutes. The column effluent was introduced into the ion source of a Pegasus BT time-of-flight mass spectrometer, GC/TOFMS (Leco Corp., St Joseph, MI, USA). The transfer line and the ion source temperatures were 250 °C and 200 °C, respectively. Ions were generated by a 70 eV electron beam at an ionization current of 2.0 mA, and 30 spectra s-1 were recorded in the mass range m/z 50 - 800. The acceleration voltage was turned on after a solvent delay of 150 seconds. The detector voltage was 1800-2300 V.

Data Analysis: All non-processed MS-files from the metabolic analysis were exported from the ChromaTOF software in NetCDF format to MATLAB R2021a (Mathworks, Natick, MA, USA), where all data pre-treatment procedures, such as base-line correction, chromatogram alignment, data compression and Multivariate Curve Resolution were performed. The extracted mass spectra were

identified by comparisons of their retention index and mass spectra with libraries of retention time indices and mass spectra (Schauer et al 2005). Mass spectra and retention index comparison was performed using NIST MS 2.2 software. Annotation of mass spectra was based on reverse and forward searches in the library. Masses and ratio between masses indicative of a derivatized metabolite were especially notified. The mass spectrum with the highest probability indicative of a metabolite and the retention index between the sample and library for the suggested metabolite was ± 5 (usually less than 3) the deconvoluted "peak" was annotated as an identification of a metabolite.

<u>Hormone Analysis:</u> Samples were extracted, purified, and analyzed according to method the described in Šimura et al., (2018), with slight modifications. Briefly, approximately 20 mg of frozen material per sample was homogenized and extracted in 1 mL of ice-cold 60% aqueous acetonitrile (v/v) with a mixture of 13C- or deuterium-labelled internal standards using three carbide beads in a bead mill (27 hz, 10 min, 4°C; MixerMill, Retsch GmbH, Haan, Germany). After centrifugation (14 000 RPM, 15 min, 4°C), the supernatant was purified as follows. A solid-phase extraction column, Oasis HLB (30 mg 1 cc, Waters Inc., Milford, MA, USA) was conditioned with 1ml of 100% methanol and 1ml of deionized water (Milli-Q, Merck Millipore, Burlington, MA, USA). After the conditioning steps each sample was loaded onto the SPE column and flow-through fraction was collected with the elution fraction, 1ml 30% aqueous acetonitrile (v/v). Samples were evaporated to dryness using a speed vac (SpeedVac SPD111V, Thermo Scientific, Waltham, MA, USA). Prior LC-MS analysis, samples were dissolved in 10 µL ACN followed by 40 µl H20 and transferred to insert-equipped vials. The analysis was performed on a UHPLC-ESI-MS/MS system comprising of an Agilent 1290 Infinity Binary LC System coupled to a 6495 Triple Quad LC/MS System with Jet Stream and Dual Ion Funnel technologies (Agilent Technologies, Santa Clara, CA, USA). Chromatic separation was achieved by injecting 15 µl of each sample were injected onto a Acquity UPLC CSH 150 m x 2.1 mm, 1.7 µm C18 RP column held at 40 °C. The gradient elution buffers were A (H2O, 0.01 % formic acid) and B (ACN, 0.01 % formic acid), and the flow-rate was 0.25 mL min-1. The compounds were eluted with a gradient consisting of 5 % B over 10 minutes, B was increased to 80 % over 20 minutes and held at 80 % for 1 minute; B was decreased to 5 % for 0.5 minutes and held for 2.5 minutes. The MS parameters were optimized for each compound as described in Table X. MRM transitions for the derivatized amino acids were optimized using MassHunter MS Optimizer software (Agilent Technologies Inc., Santa Clara, CA, USA). The quantification was carried out in the Agilent MassHunter Workstation Software Quantitative (Agilent Technologies, Santa Clara, CA, USA).

Table 1. Hormones, their retention times and MRM detection masses

Name	Short Name	RT (min)	MRM transition	IS	MRM transition	MRM mode
Tryptophan	Trp	5,48	205,2>146,1	d5-Trp	210,2>150,1	+
Tryptamin	Tra	3,15	161,1>144,1	d2-Tra	165,1>148,1	+
Anthranilic acid	ANT	15,70	138,1>120,1	13C6-ANT	142,1>124,1	+
oxIAA-glucose	oxIAA-Glc	14,55	192,1>146,1	13C6-oxIAA-Glc	198,2>152,1	+
Indole-3-acetamide	IAM	15,86	175,1>130,1	d5-IAM	180,2>134,1	+
IAA-Aspartate	IAAsp	17,37	291,2>130,1	13C6-IAAsp	297,2>136,1	+
2-oxindole-3-acetic acid	oxIAA	16,00	192,1>146,1	13C6-oxIAA	198,2>152,1	+
IAA-Glucose	IAA-Glc	16,52	176,6>130,1	13C6-IAA-Glc	182,1>136,1	+
IAA-Glutamate	IAGlu	17,60	305,2>130,1	13C6-IAGlu	311,2>136,1	+
Indole-3-acetic acid	IAA	18,90	176,6>130,1	13C6-IAA	182,1>136,1	+
Indole-3-acetonitrile	IAN	21,18	157,1>130,1	d4-IAN	161,2>134,1	+
trans-zeatin	tZ	5,04	220,1>130,1	13C5-tZ	225,1>141,1	+
cis-zeatin	cZ	6,51	220,1>130,1	13C5-cZ	225,1>141,1	+
Isopentenyladenine	iP	15,59	204,2>136,1	d6-iP	210,2>137,1	+
Dihydrozeatin	DHZ	5,16	222,1>136,1	d3-DHZ	225,1>136,1	+
trans-zeatin riboside	tZR	14,61	352,2>220,1	d5-tZR	357,2>225,1	+
cis-zeatin riboside	cZR	14,94	352,2>220,1	d5-tZR	357,2>225,1	+
Isopentenyladenosine	iPR	17,82	336,3>204,1	d6-iPR	342,3>210,1	+
Dihydrozeatin riboside	DHZR	14,66	354,2>222,1	d3-tZR	357,2>225,1	+
Jasmonic acid	JA	21,84	209,2>58,8	d6-JA	215,2>58,8	-
Jasmonoyl-L-isoleucine	JA-Ile	23,80	324,3>151,2	d2-JA-Ile	326,3>151,2	+
cis-12-oxo-phytodienoic acid	cisOPDA	27,78	293,3>275,3	d5-cisOPDA	298,3>279,3	+
Salicylic acid	SA	20,72	137,1>92,8	13C6-SA	143,0>98,8	-
Gibberellin A1	GA1	17,44	347,1>259,1	d2-GA1	349,1>261,1	-
Gibberellin A3	GA3	17,35	345,1>239,1	d2-GA1	349,1>261,1	-
Gibberellin A4	GA4	23,23	331,1>257,1	d2-GA4	333,1>259,1	-
Gibberellin A7	GA7	23,09	329,1>223.1	d2-GA4	333,1>259,1	-
Brassinolide	BL	24,87	481,1>445,1	d3-BL	484,1>448,1	+
Castasterone	CS	25,90	465,1>429,1	d3-CS	468,1>432,1	+

Chemicals

GC-MS chemicals:

Solvents: H20, Milli Q. Regents: Methoxy amine was purchased from Sigma (St. Louis, MO, USA), Pyridine, was purchased from Thermo Fisher Scientific (Waltham, MA, USA) and MSTFA, 1%TMCS, was purchased from Restek (Bellefonte, PA, USA). Internal standards: L-proline-13C5, alpha-ketoglutarate-13C4, myristic acid-13C3, cholesterol-D7 were obtained from Cil (Andover, MA, USA). Succinic acid-D4, salicylic acid-D6, L-glutamic acid-13C5,15N, putrescine-D4, hexadecanoic acid-13C4, D-glucose-13C6, D-sucrose-13C12 were obtained from Sigma (St. Louis, MO, USA).

Hormonomics chemicals:

Acetonitrile (ACN), hypergrade for LC-MS LiChrosolv® from Merck (Darmstadt, Germany), 60% and 30% aqueous solutions (v/v). Formic acid, Aldrich Chemical Company, Inc. (Milwaukee, WI, USA). H2O (MQ-water), purified by a Milli-Q Gradient system (Millipore, Milford, MA, USA)

Acknowledgement

Swedish Metabolomics Centre, Umeå, Sweden (www.swedishmetabolomicscentre.se) is acknowledged for metabolic profiling by GC-MS and plant hormone analysis by LC-QqQ-MSMS.

References

Šimura, J., Antoniadi, I., Široká, J., Tarkowská, D., Strnad, M., Ljung, K., & Novák, O. (2018). Plant Hormonomics: Multiple Phytohormone Profiling by Targeted Metabolomics. Plant Physiology, 177(2), 476–489.

Gullberg J, Jonsson P, Nordström A, Sjöström M & Moritz T. Design of experiments: an efficient strategy to identify factors influencing extraction and derivatization of Arabidopsis thaliana samples in metabolomic studies with gas chromatography/mass spectrometry. Anal Biochem 2004 331 283-295.

Schauer N, Steinhauser D, Strelkov S, Schomburg D, Allison G, Moritz T, Lundgren K, Roessner-Tunali U, Forbes MG, Willmitzer L, Fernie AR & Kopka J. GC-MS libraries for the rapid identification of metabolites in complex biological samples. FEBS Lett 2005 579 1332-1337.
